# Gender correlation between sleep duration and risk of coronary heart disease: a systematic review and meta-analysis

**DOI:** 10.3389/fcvm.2025.1452006

**Published:** 2025-03-25

**Authors:** Cun Li, Shun-xin Luo, Tian-wei Liang, Dan Song, Jin-xiao Fu

**Affiliations:** ^1^ School of Clinical Medicine, Dali University, Dali, Yunnan, China; ^2^ Geriatric Department, Affiliated Hospital of Yunnan University, Kunming, Yunnan, China

**Keywords:** extreme sleep duration, coronary heart disease, gender, BMI, continent

## Abstract

**Objective:**

The influence of extreme sleep duration on coronary heart disease (CHD) risk across genders remains a debated topic.

**Methods:**

This analysis gathers observational studies that explore association between varying sleep durations and CHD risks. Trend estimation employs generalized least squares, converting specific category risk estimates into relative risks (RR) per hour of sleep increase. A two-stage hierarchical regression model evaluates potential linear dose-response relationships. Data analysis utilizes random-effects restricted cubic spline models with four knots.

**Results:**

Involving 17 studies and 906,908 participants, this meta-analysis identifies a pronounced U-shaped nonlinear relationship between sleep duration and CHD risk applicable to both genders (*P* < 0.01). Notably, shorter sleep durations are linked to higher CHD risks in women, whereas longer durations are more consequential for men. The optimal sleep duration for minimizing CHD risk is between 7.0–8.0 h daily for men and 7.5–8.5 h for women.

**Conclusion:**

The influence of sleep duration on CHD risk differs significantly between genders.

**Systematic Review Registration:**

https://www.crd.york.ac.uk/PROSPERO/myprospero, identifier (CRD42023478235).

## 1 Introduction

Cardiovascular diseases (CVD), including congestive heart failure (CHF), coronary heart disease (CHD), angina pectoris (AP), myocardial infarction, and stroke, pose major public health challenges and are key mortality causes globally (1). The WHO Global Burden of Disease Project highlights that coronary artery disease (CAD) caused one million deaths in East Asia and the Pacific Region, with 8.2 million angina cases and 11.8 million Disability-Adjusted Life Years (DALYs) attributed to CAD in this area (2). Stroke and CAD account for 60%–70% of CVD mortality in China (2, 3). In the U.S., around 720,000 individuals annually experience acute myocardial infarction (MI) or die from CAD, with over 335,000 facing recurrent episodes (4). Notably, older adults (aged 75 and above) form a significant portion (30%–40%) of acute coronary syndrome (ACS) hospitalizations and suffer the highest ACS-related mortality (5–7). This demographic often presents complex clinical pictures, including extensive atherosclerotic plaque, anatomical complexities, calcifications, vessel tortuosity, ostial lesions, multivessel disease, left main stenosis and concurrent geriatric syndromes, raising their overall risk (8).

Although CHD mortality rates have marginally declined, it remains a leading death cause. Risk factors for CAD fall into non-modifiable (age, gender, ethnicity, family history) and modifiable categories (hypertension, hyperlipidemia, diabetes, obesity, smoking, poor diet, sedentary lifestyle, stress) (9–13). Gender is an immutable risk factor, whereas sleep duration is a modifiable one. Both have been extensively examined for their effects on health outcomes (14). Research shows notable gender disparities in the incidence and timing of CHD due to physiological, psychological, and other factors (15). Shift work, active family responsibilities, and extensive use of electronic devices lead to prevalent short and long sleep durations across gender groups (16). Sleep plays a crucial role in the development and progression of cardiovascular diseases (17).

Previous studies have shown a U-shaped relationship between CHD and sleep duration across genders (18, 19). Yet, a 2018 study on cardiovascular diseases, despite its recency and breadth, did not isolate gender in its analysis, revealing a diminished link between short sleep durations and adverse events post adjustment for various factors (20). This observation suggests potential oversight in gender-specific research in earlier studies. Moreover, literature reviews indicate that after comprehensive adjustments, varying sleep durations differently affect cardiovascular disease outcomes by gender. Several studies associate short sleep durations exclusively with increased CHD risks in women (21–25), whereas others suggest similar associations in men alone after such adjustments (26, 27). Svensson's findings contradict earlier studies, showing similar correlations between short sleep durations and CHD risks in both genders (28), while Kronholm's research suggests no link between excessive sleep durations and CHD risks in males (29). Sex-specific differences may exist in the association between extreme sleep duration and CHD incidence. These differences could be attributed to distinct hormonal patterns and metabolic processes, reflecting inherent pathophysiological dimorphism in the stratification of cardiovascular risk across genders. Most recent meta-analyses have not included gender subgroup analyses (20), and those that did are considered outdated (18, 19). Since 2016, new research examining sleep durations and CHD risks by gender has emerged (23–25, 28, 30, 31). Our study incorporates this recent literature to explore potential U-shaped or J-shaped relationships between CHD and sleep duration among different genders and the differences therein. These findings have significant health implications and may inform future healthcare policies.

## 2 Methods

The study adhered to the Preferred Reporting Items for Systematic Reviews and Meta-Analyses (PRISMA) guidelines for reporting items and their requirements for network meta-analysis (NMA). The meta-analysis was conducted in compliance with these guidelines. The study protocol is registered in the International Prospective Register of Systematic Reviews (Registration Number: CRD42023478235).

### 2.1 Literature search strategy and selection criteria

Searches in PubMed, Embase, Cochrane Library, and Web of Science were conducted until October 2023 using unrestricted terms related to sleep coronary artery disease, coronary artery diseases, coronary disease, coronary diseases, coronary heart disease, coronary heart diseases, multivessel coronary artery disease (details in Supplementary File). This search also included a review of studies from previous meta-analyses to identify additional relevant literature. Inclusion Criteria: (1) Observational studies examining the association between sleep duration and incident risk of CHD; (2) Studies reporting relative risk (RR) with 95% confidence intervals (CIs) for different sleep durations; (3) Studies focusing on the correlation of sleep duration with CHD risk across various genders. Exclusions applied to case reports, guidelines, letters, chapters, conference abstracts, editorials, meta-analyses, reviews, and animal experiments.

### 2.2 Data extraction

Title and abstract screening were independently performed by two authors to identify relevant studies. Discrepancies were resolved through discussion or consultation with a third author. Full-text articles were then assessed against the selection criteria for final inclusion. If necessary, further information or original data were requested from study authors (first author, corresponding author, or the authors' department). Included articles contained RR values with 95% CIs for both genders and various sleep durations related to CHD risk. Data extracted included author, year, sample size, study period, type, geographical information, average age, body mass index, gender distribution, sleep groups, RR values, and follow-up duration, compiled in an Excel spreadsheet.

### 2.3 Assessment of study quality

The quality of cohort and case-control studies will be assessed using the Newcastle Ottawa Scale (NOS). The NOS conducts a comprehensive evaluation from three aspects of the study: selection, comparability, and outcome (cohort studies) or exposure (case-control studies) (32, 33). Each study can earn up to one point per item in the selection and exposure categories and up to two points for comparability (Tables 1, 2). Studies will be classified as low (0–3), moderate (4–6), or high quality (7–9). For cross-sectional studies, quality assessment will use the Agency for Healthcare Research and Quality (AHRQ) 11-item checklist. Each item scores “1” for “YES” and “0” for “UNCLEAR” or “NO.” Quality categories are low (0–3), moderate (4–7), and high (8–11) (Table 3).

**Table 1:** Newcastle-Ottawa scale (cohort) for five studies included in this meta-analysis.

Study	Selection				Comparability	Outcome			Quality scores
	Representativeness of the exposed cohort	Selection of the nonexposed cohort	Ascertainment of exposure	Demonstration that outcome of interest was not present at start of study	Comparability of cohorts on the basis of the design or analysis	Assessment of outcome	Was follow-up long enough for outcomes to occur	Adequacy of follow up of cohorts	
Amagai et al. (2010) (26)	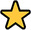	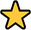		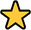	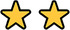	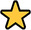	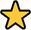	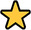	8
Ayas et al. (2003) (86)		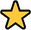	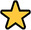	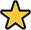	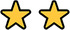	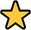	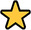		7
Chandola et al. (2010) (27)		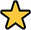	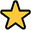	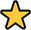	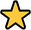	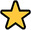	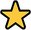		6
Ikehara et al. (2009) (22)		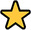		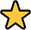	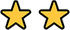	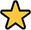	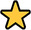		6
Meisinger et al. (2007) (21)	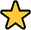	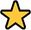		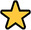	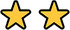		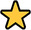	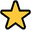	7
Sands-Lincoln et al. (2013) (87)		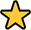	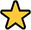	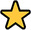	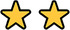	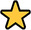	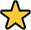		7
Svensson et al. (2018) (28)	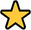	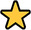		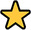	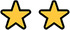	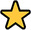	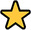		7
Hamazaki et al. (2011) (34)		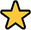		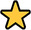	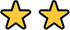	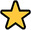	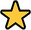		6
Khan et al. (2018) (35)	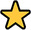	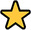		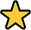	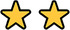	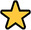	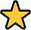	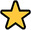	8
Kronholm (2011) (29)	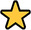	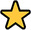		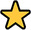	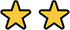	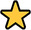	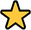		7
Lao (2018) (23)		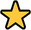	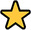	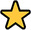	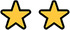		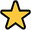		6
Li (2013) (36)		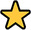		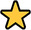	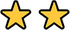	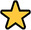	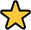	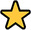	7
Shankar (2008) (18)		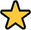	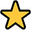	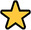	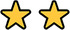	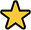	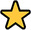	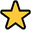	8
Strand (2016) (30)		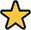	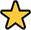		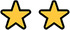	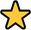	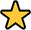		6

**Table 2:** Newcastle-Ottawa scale (case-control) for ten studies included in this meta-analysis.

Study	Selection				Comparability	Exposure			Overall
	Is the case definition adequate?	Representativeness of the cases	Selection of controls	Definition of Controls	Comparability of cohorts on the basis of the design or analysis	Ascertainment of exposure	Same method of ascertainment for cases and controls	Non-response rate	
Cheng (2014) (37)	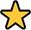	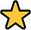	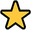	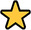	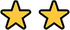		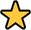		7
Li (2017) (38)		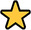	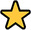	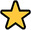	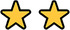		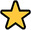		6
Zhang (2021) (39)	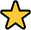	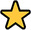	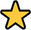	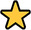	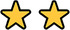		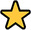		7

**Table 3:** Agency for healthcare research and quality (AHRQ) checklist (cross-sectional) for 5 studies included in this meta-analysis.

Agency for healthcare research and quality (AHRQ) checklist (cross-sectional) for 5studies included in this meta-analysis	Sabanayagam 2010 (19)	Yazdanpanah 2020 (24)	Kadier 2022 (1)	Suzuki 2018 (31)	Petrovic 2020 (40)
Item					
(1) Define the source of information (survey, record review)	1	1	1	1	1
(2) List inclusion and exclusion criteria for exposed and unexposed subjects (cases and controls) or refer to previous publications	0	1	1	1	1
(3) Indicate time period used for identifying patients	1	1	1	1	1
(4) Indicate whether or not subjects were consecutive if not population-based	0	1	0	0	0
(5) Indicate if evaluators of subjective components of study were masked to other aspects of the status of the participants	1	1	1	1	1
(6) Describe any assessments undertaken for quality assurance purposes (e.g., test/retest of primary outcome measurements)	1	0	0	0	1
(7) Explain any patient exclusions from analysis	0	1	1	1	1
(8) Describe how confounding was assessed and/or controlled	1	1	1	1	1
(9) If applicable, explain how missing data were handled in the analysis	1	0	1	1	0
(10) Summarize patient response rates and completeness of data collection	0	0	0	0	0
(11) Clarify what follow-up, if any, was expected and the percentage of patients for which incomplete data or follow-up was obtained	1	1	1	1	1
Total score	7	8	8	8	8

### 2.4 Statistical analysis

This analysis will utilize multivariate-adjusted RRs with 95% CIs. Pooled RRs and CIs will be estimated using a fixed-effects model for *I*² < 50% and *p* > 0.1; otherwise, a random-effects model is applied. Significant heterogeneity will lead to further subgroup analyses, focusing on BMI, age, region, and study type. Subgroup heterogeneity *I*² < 50% will indicate a major source of data variability. Sensitivity analysis will remove studies one by one to check the influence on overall results. Egger's test will assess potential publication bias. Dose-response analysis will be based on sleep duration categories, case numbers, total cases, and the logarithm of RRs and standard errors. Studies must provide data across at least three exposure categories, with median sleep durations assigned to each. For open-ended upper categories, the amplitude is assumed equal to the adjacent lower category. Using the generalized least-squares method for trend estimates, category-specific risk estimates will be converted into relative risk (RR) for each additional hour of sleep duration. A two-stage hierarchical regression model will assess the linear dose-response relationship between sleep duration and CHD, with data modeled using random-effects restricted cubic spline models with four knots.

## 3 Results

### 3.1 Literature search

**Figure 1:**
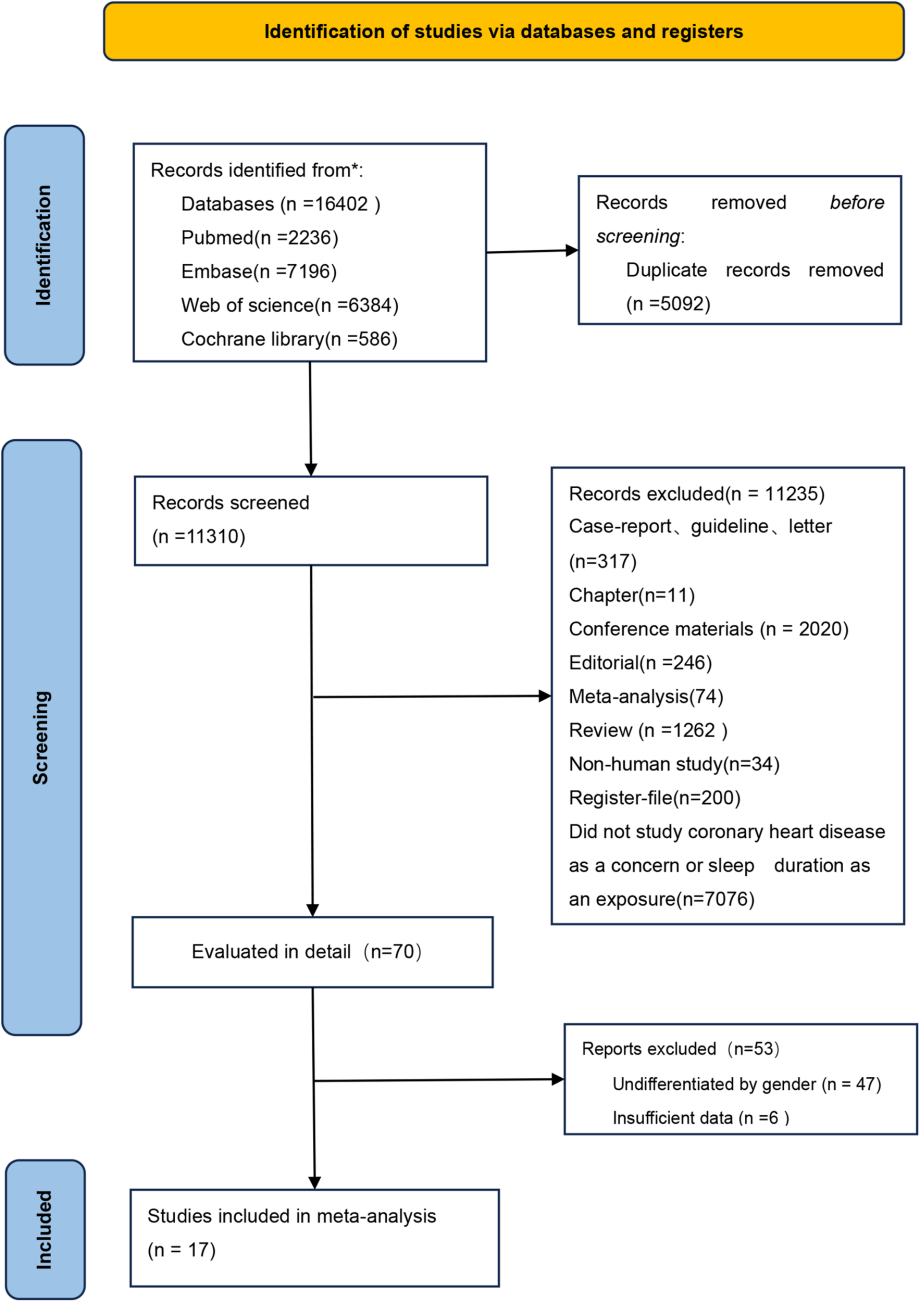
Flowchart of the selection process for eligible studies (PRISMA).

The study identification and inclusion process are depicted in Figure 1. Searches yielded 2,236 articles from PubMed, 7,196 from Embase, 586 from Cochrane Library, and 6,387 from Web of Science as of October 11, 2023. After removing 5,092 duplicates, 11,310 articles were screened by title and abstract. Of these, 11,235 were excluded for being irrelevant, such as case reports or conference materials. Detailed full-text review of 70 articles led to the exclusion of 47 due to lack of gender differentiation, and five more (1, 35, 36, 38, 40) for insufficient data. Ultimately, 17 articles were selected for the meta-analysis.

### 3.2 Study characteristics

The key data from the included studies are summarized in the provided Table 4. These studies, published between 2003 and 2021, comprise 12 cohort studies, 2 case-control studies, and 3 cross-sectional studies. Geographical distribution included 9 studies in Asia, 5 in Europe, and 3 in North America. Sample sizes ranged from 966 to 392,164, totaling 906,908 participants. All studies, except (27), adjusted for various covariates. The mean age of male participants was 41.78–64.10 years, and for females, it was 41.78–63.36 years. BMI information was provided in all but two studies (25, 28), ranging from 22.62 to 29.60 in men and 22.90–29.59 in women. The primary focus was CHD, although some studies also included stroke patients (19, 26, 29, 34). Follow-up periods varied from 1.2 to 34 years. The quality assessment scores ranged from 6 to 8, averaging 6.94, with cohort studies averaging 6.75, cross-sectional studies 7.67, and case-control studies 7.

**Table 4:** Basic characteristics of included studies.

Study	Country	Research method	Data collection time	No of participants	Age	M/F	BMI	Outcome	Methods of obtaining sleep duration	Race	Follow-up year	Quality assessment
Amagai et al. (2010) (26)	Japan	Cohort	1,992.4-1,995.7	11,367	Male: 55.089 ± 11.93 Female: 55.29 ± 11.21	4,413/6,954	Male: 22.93 ± 2.87 Female: 23.18 ± 3.19	CHD, stroke	Survey	East Asian	10.7 years	8
Zhang et al. (2021) (39)	China	Case-control	NA	27,935	55.96 ± 12.22	11,177/16,758	NA	CHD	Survey	East Asian	NA	7
Sands-Lincoln et al. (2013) (87)	The United States	Cohort	1,993-1,998	86,329	63.36 ± 7.34	All females:86,329	Female: 27.12 ± 5.79	CHD	Survey	/	10.3 years	7
Sabanayagam et al. (2010) (19)	The United States	Cross-sectional	2,005	30,397	47.59 ± 17.76	13,452/16,945	29.59 ± 14.05	CHD, stroke	Survey	/	NA	7
Meisinger et al. (2007) (21)	Germany	Cohort	1,984–1,995	6,896	Male: 57.54 ± 8.03 Female: 57.47 ± 8.07	3,508/3,388	Male: 27.81 ± 3.52 Female: 27.62 ± 4.71	CHD	Survey	/	10.1 years	7
Ikehara et al. (2009) (22)	Japanese	Cohort	1,988–1,990	98,634	Males: 54.43 Females: 57.35	41,489/57,145	Male: 22.62 Female: 22.90	CHD	Survey	East Asian	14.3 years	6
Chandola et al. (2010) (27)	England	Cohort	1,985–1,988	9,841	44.45	6,601/3,240	24.63	CHD	Survey	/	15 years	6
Ayas et al. (2003) (86)	The United States	Cohort	1,986	71,617	All females: 52.21	All female:71,617	All females: 24.75	CHD	Survey	/	10 years	7
Yazdanpanah et al. (2020) (24)	Iranian	Cross-sectional	2,015–2,016	10,129	Male: 48.61 ± 9.61 Females: 48.64 ± 9.54	4,575/5,554	Male: 24.15 ± 4.52 Female: 26.86 ± 4.81	CHD	Survey	/	NA	8
Svensson et al. (2018) (28)	Sweden	Cohort	1,991–1,996	16,344	Male: 57.28 ± 5.98 Females: 57.27 ± 6.03	6,966/9,378	NA	CHD	Survey	/	NA	7
Strand et al. (2016) (30)	Taiwan, China	Cohort	1,994–2,011	392,164	41.78 ± 14.24	191,656/200,508	23.02 ± 3.58	CHD mortality	Survey	East Asian	9.7years	6
Shankar et al. (2008) (18)	Singapore	Cohort	1,993–1,998	58,044	56.37 ± 0.96	25,552/32,492	23.02 ± 0.17	CHD mortality	Survey	East Asian	14years	8
Lao et al. (2018) (23)	Taiwan, China	Cohort	1,996–2,014	57,846	50.6 ± 8.6	26,752/31,094	23.8 ± 3.2	CHD	Survey	East Asian	5.6years	6
Kronholm et al. (2011) (29)	Finland	Cohort	1,972–1,977	23,290	Male: 42.39 ± 10.51 Female: 43.49 ± 10.59	11,373/11,917	Male:25.82 ± 3.45 Female:26.20 ± 4.66	CHD, stroke	Survey	/	29–34years	7
Hamazaki et al. (2011) (34)	Japan	Cohort	1,994	2,282	43.7 ± 5.5	All male: 2,282	All males:22.9 ± 2.7	Stroke, coronary events and sudden cardiac death	Survey	East Asian	14years	6
Suzuki et al. (2018) (31)	Japan	Cross-sectional	2,006–2,008	1,093	64.1 ± 9.9	All male: 1,093	23.6 ± 3.0	CAC	Survey	East Asian	NA	8
Cheng et al. (2014) (37)	Taiwan, China	Case-control	2,008–2,011	966	51.4 ± 7.03	All male: 966	25.03 ± 3.294	CHD	Survey	East Asian	NA	7

### 3.3 Statistical analysis results

**Figure 2:**
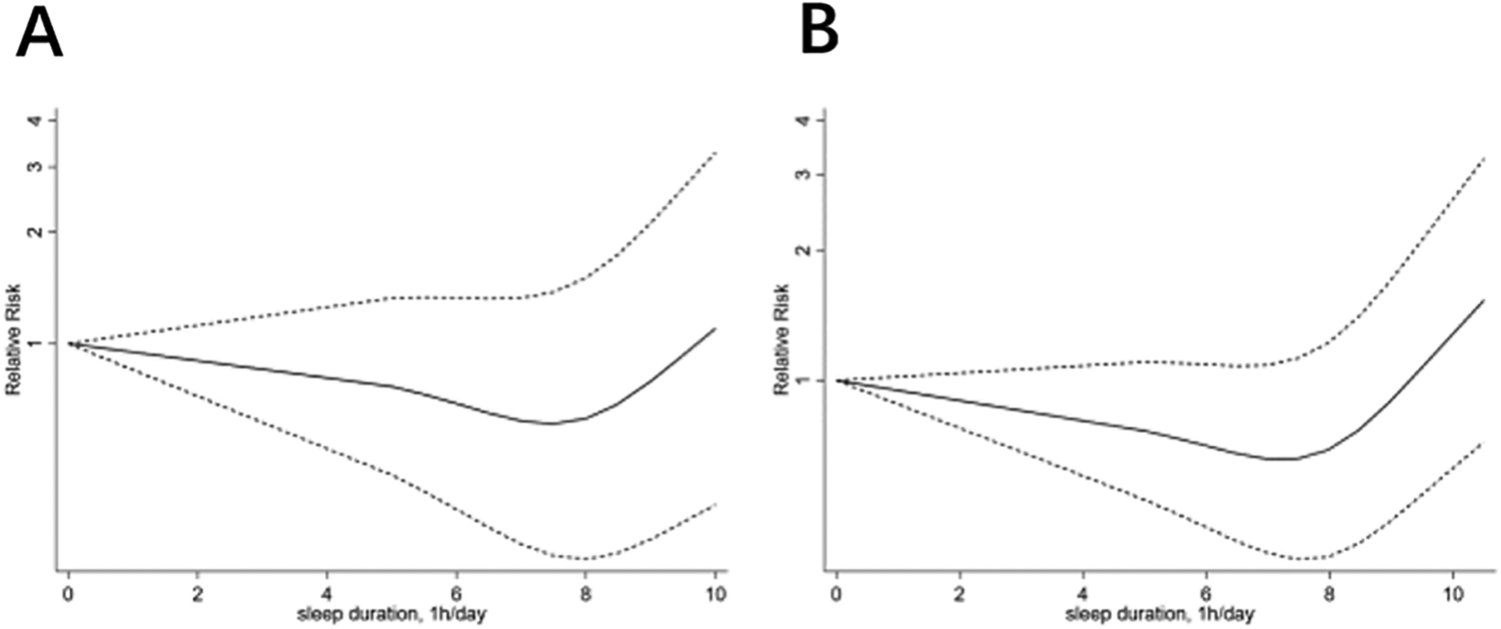
The dose-response analysis of coronary heart disease and sleep duration, **(A)** for woman, **(B)** for man.

This meta-analysis included 15 studies focusing on male participants, totaling 351,855 individuals. The dose-response analysis indicated a significant non-linear correlation between sleep duration and CHD risk in males (*P* < 0.01). The lowest CHD risk was observed at a sleep duration of 7.0–8.0 h per day (Figure 2B). In the heterogeneity test, the *I*² value was 80.9% when comparing the highest to normal sleep duration (*P* < 0.01). A random-effects model showed that the highest sleep duration group had a significantly increased CHD risk, with a combined RR of 1.35 (95% CI: 1.15, 1.58, *P* < 0.01) (Supplementary Figure S14). Similarly, the lowest sleep duration compared to normal sleep duration showed a heterogeneity *I*² of 93.3% (*P* < 0.001). Here, the lowest sleep duration group also exhibited a higher CHD risk, with a combined RR of 1.52 (95% CI: 1.16, 2.01, *P* < 0.01) (Supplementary Figure S9). Despite substantial heterogeneity, subgroup analyses based on study type (cohort study, cross-sectional study, case-control study), continent (Europe, Asia, North America), average age (age ≥55 years, age <55 years), and BMI (BMI ≥24, BMI <24) did not pinpoint its source. However, these analyses yielded several interesting findings, as detailed in the following sections.

In the subgroup analysis comparing the highest sleep duration to normal sleep duration in males, cohort studies showed an increased risk of CHD with an RR of 1.24 (95% CI: 1.10–1.41, *P* < 0.01). However, this increase was not observed in cross-sectional and case-control studies (Cross-sectional: *P* = 0.122, Case-control: *P* = 0.089) (Supplementary Figure S17). In the analysis by continent, the highest sleep duration was associated with an increased CHD risk in Europe (RR: 1.14, 95% CI: 1.01–1.28, *P* = 0.03), Asia (RR: 1.35, 95% CI: 1.16–1.57, *P* < 0.01), and North America (RR: 2.55, 95% CI: 2.11–3.09, *P* < 0.01) (Supplementary Figure S18). Age-based subgroup analysis revealed that males aged ≥55 years (RR: 1.34, 95% CI: 1.15–1.57, *P* < 0.01) and <55 years (RR: 1.37, 95% CI: 1.15–1.58, *P* = 0.014) both showed increased risk with the highest sleep duration (Supplementary Figure S16). In the BMI subgroup analysis, males with BMI ≥24 did not show increased CHD risk with the highest sleep duration (*P* = 0.056), whereas those with BMI <24 did (RR: 1.30, 95% CI: 1.11–1.52, *P* < 0.01) (Supplementary Figure S15).

In the subgroup analysis of study types, cohort studies showed an increase in CHD risk with the lowest sleep duration in males compared to normal (RR: 1.44, 95% CI: 1.20–1.73, *P* < 0.01). However, cross-sectional and case-control studies did not demonstrate a significant risk increase (Cross-sectional: *P* = 0.201, Case-control: *P* = 0.235) (Supplementary Figure S12). In the continent subgroup analysis, Asian males showed no significant association between the lowest sleep duration and CHD risk (*P* = 0.084). Conversely, European males (RR: 1.43, 95% CI: 1.27–1.62, *P* < 0.01) and North American males (RR: 2.17, 95% CI: 1.77–2.67, *P* < 0.01) exhibited increased CHD risk with the lowest sleep duration (Supplementary Figure S13). Age subgroup analysis indicated that for both age groups ≥55 and <55, the lowest sleep duration increased CHD risk (RR: 1.62, 95% CI: 1.23–2.14, *P* < 0.01 for ≥55; RR: 1.68, 95% CI: 1.22–2.32, *P* < 0.01 for <55) (Supplementary Figure S11). Finally, in the BMI subgroup analysis, males with the lowest sleep duration showed increased CHD risk regardless of BMI being ≥24 (RR: 1.79, 95% CI: 1.18–2.72, *P* < 0.01) or <24 (RR: 1.32, 95% CI: 1.01–1.72, *P* = 0.042) (Supplementary Figure S10).

In this meta-analysis, 14 articles focusing on 553,319 female participants were included. The dose-response analysis showed a significant non-linear association (*P* < 0.01) between sleep duration and CHD risk in females. The optimal sleep duration for the lowest CHD risk was 7.5–8.5 h per day (Figure 2A). High heterogeneity was observed in the comparison between the highest and normal sleep durations (*I*² = 87.8%, *P* < 0.01), with a significant risk increase indicated by a combined RR of 1.36 (95% CI: 1.10–1.69, *P* = 0.005) (Supplementary Figure S24). Similarly, the comparison between the lowest and normal sleep durations showed high heterogeneity (*I*² = 90.3%, *P* < 0.001) and a significant risk increase (combined RR: 1.66, 95% CI: 1.35–2.06, *P* < 0.01) (Supplementary Figure S19). Despite high heterogeneity, subgroup analysis did not pinpoint its source but revealed several notable findings.

In the female cohort studies, the highest sleep duration compared to normal increased CHD risk (RR: 1.33, 95% CI: 1.14–1.54, *P* < 0.01). However, cross-sectional and case-control studies did not show a significant risk increase (Cross-sectional: *P* = 0.409, Case-control: *P* = 0.134) (Supplementary Figure S27). Continent-wise, European and North American females showed no significant risk association (European: *P* = 0.257, North American: *P* = 0.056), while Asian females exhibited increased risk (RR: 1.29, 95% CI: 1.08–1.55, *P* < 0.01) (Supplementary Figure S28). Age subgroup analysis showed increased risk in females aged ≥55 (RR: 1.29, 95% CI: 1.14–1.46, *P* < 0.01), but no significant association in females aged <55 (*P* = 0.066) (Supplementary Figure S26). In the BMI subgroup analysis, females with BMI ≥24 showed no significant risk association (*P* = 0.084), whereas those with BMI <24 had increased risk (RR: 1.34, 95% CI: 1.05–1.71, *P* = 0.018) (Supplementary Figure S25).

In the female cohort studies, the lowest sleep duration compared to normal was linked to an increased CHD risk (RR: 1.55, 95% CI: 1.26–1.91, *P* < 0.01). This pattern was also seen in case-control studies (RR: 2.12, 95% CI: 1.50–2.99, *P* < 0.01), but not in cross-sectional studies (*P* = 0.097) (Supplementary Figure S22). In continent-based subgroup analysis, European females (RR: 1.73, 95% CI: 1.24–2.41, *P* < 0.01) and Asian females (RR: 1.58, 95% CI: 1.21–2.05, *P* < 0.01) with the lowest sleep duration had increased CHD risk, unlike North American females (*P* = 0.145) (Supplementary Figure S23). Age-based subgroup analysis showed increased CHD risk in females aged ≥55 (RR: 1.62, 95% CI: 1.23–2.14, *P* < 0.01) and <55 (RR: 1.68, 95% CI: 1.22–2.32, *P* < 0.01) with the lowest sleep duration (Supplementary Figure S21). BMI-based subgroup analysis indicated that females with BMI ≥24 (RR: 1.74, 95% CI: 1.22–2.47, *P* < 0.01) and <24 (RR: 1.41, 95% CI: 1.12–1.77, *P* < 0.01) had increased CHD risk with the lowest sleep duration (Supplementary Figure S20).

### 3.4 Sensitivity analysis and publication bias

In the sensitivity analysis, excluding low-quality studies sequentially did not significantly change the combined RR values (Supplementary Figure S5–S8). Extensive Egger regression tests were conducted to check for publication bias. The results indicated no evidence of potential publication bias (*P* = 0.565 for men with short sleep duration and CHD; *P* = 0.956 for men with long sleep duration and CHD; *P* = 0.125 for women with short sleep duration and CHD; *P* = 0.632 for women with long sleep duration and CHD) (Supplementary Figure S1–S4).

## 4 Discussion

This meta-analysis reveals a significant non-linear U-shaped association between sleep duration and CHD risk for both genders (both *P* < 0.01). Notably, short sleep duration impacts females' CHD risk more significantly, whereas long sleep duration affects males more. The ideal sleep duration is about 7.0–8.0 h daily for males and 7.5–8.5 h for females.

Sleep deprivation promotes CAD through complex pathophysiological mechanisms. A primary pathway involves elevated reactive oxygen species (ROS) (41). These ROS facilitate the oxidation of low-density lipoprotein (LDL) into oxidized LDL (ox-LDL). The resulting ox-LDL undergoes uptake by macrophages through scavenger receptors, leading to foam cell formation. These foam cells constitute essential components of atherosclerotic plaques and release proinflammatory cytokines, creating a pathogenic inflammatory cycle (42). ROS overproduction compromises endothelial function through multiple pathways. This process includes impairment of eNOS function (43), resulting in decreased nitric oxide availability. The activation of MAPK and NF-κB signaling pathways by ROS disrupts normal endothelial cell processes and accelerates atherogenesis (39, 44–47). The presence of ROS compromises plaque stability through dual mechanisms: inducing apoptosis in vascular smooth muscle cells which thins fibrous caps, and activating matrix metalloproteinases (MMPs). These MMPs degrade collagen in the extracellular matrix, undermining the structural integrity of plaques (48, 49). Sleep deprivation increases interleukin-6 (IL-6) levels (50). This elevation enhances the risk of CAD through multiple pathways: stimulating hepatic C-reactive protein (CRP) production, promoting LDL oxidation through oxidative stress (51), and increasing the expression of adhesion molecules (VCAM-1/ICAM-1). These changes facilitate monocyte infiltration and subsequent foam cell development (45). Research in both animals and humans demonstrates that sleep loss reduces vagal tone (52, 53). This reduction diminishes acetylcholine-mediated suppression of proinflammatory cytokines through cholinergic receptors (54). Simultaneously, enhanced sympathetic activity increases heart rate and vascular resistance, leading to elevated blood pressure (55). Sleep deprivation disrupts leptin-ghrelin balance, characterized by decreased leptin and increased ghrelin levels. These changes promote adipocyte lipid storage and insulin resistance (56–58). The reduction in slow-wave sleep (SWS) decreases gonadotropin-releasing hormone-mediated inhibition of the hypothalamic-pituitary-adrenal axis. This decrease, combined with reduced cardioprotective growth hormone release, further compounds the risk of CAD.

Research on prolonged sleep duration and CAD remains limited. Studies in Spanish populations have shown that extended sleep is associated with reduced physical activity, increasing the risk of obesity (59)—a known risk factor for CAD. Longer sleep duration correlates with poorer sleep quality (60), elevating IL-6 and high-sensitivity CRP (hsCRP) levels, which contribute to CAD development. Prolonged sleep may also slow blood flow and increase blood viscosity, promoting thrombosis and CAD events (61). In males, the relationship may involve testosterone effects. While sleep restriction decreases testosterone levels, this reduced androgenic state might partially protect against coronary atherosclerosis through unknown mechanisms (62, 63).

Sex-specific mechanisms link short sleep duration and CAD. Females show greater leptin reduction during sleep deprivation than males, increasing weight gain susceptibility (64). Obese females have higher inflammatory marker levels than males (65), potentially explaining increased risk of CAD in women with short sleep. Rodent studies demonstrate that sleep restriction causes more prominent glucose intolerance in female mice (66), possibly due to sleep-induced estrogen disruption, as estrogen promotes glycogen synthesis through estrogen receptor alpha-mediated activation of AMP-activated protein kinase (67). Clinical studies show stronger blood pressure elevation in sleep-deprived females (68), with the Whitehall II study identifying short sleep as an independent hypertension risk factor only in females (69). These cardiovascular effects may result from increased hypothalamic-pituitary-adrenal axis reactivity in sleep-deficient women (70). Additionally, Carter et al. found increased muscle sympathetic nerve activity after sleep deprivation, particularly in postmenopausal women (71), suggesting the modulatory effects of estrogen on sympathetic activation. Insufficient sleep also elevates low-density lipoprotein cholesterol and hsCRP specifically in women (72, 73), creating conditions favorable for CAD development.

Different mechanisms may explain sex-specific associations between prolonged sleep and CAD. Extended sleep correlates with obesity, with stronger links between abdominal adiposity and atherosclerotic processes documented in males, potentially making sleep-prolonged males with central obesity more susceptible to CAD (74). Additionally, prolonged sleep increases blood viscosity (61), affecting males disproportionately due to their naturally higher baseline blood viscosity. This sex difference results from several factors: males have higher hematocrit levels (75), while females benefit from cardioprotective high-density lipoprotein effects (76). Higher smoking rates among males further contribute to elevated blood viscosity (77). The combined effects of physical inactivity and obesity-induced viscosity elevation likely have greater cardiovascular impact on males due to their pre-existing hyperviscous state.

Further subgroup analysis yielded intriguing findings. For instance, short sleep duration does not correlate with CHD risk in Asian males (*P* = 0.084), possibly due to an incomplete sample or lower obesity rates in this group, which diminishes the effect of CVD caused by insufficient sleep duration related to obesity (78, 79). Conversely, excessive sleep duration shows no significant association with CHD risk in European and North American females (Europe: *P* = 0.257, North America: *P* = 0.056). These disparities suggest that gender significantly influences the relationship between long sleep duration and CHD risk. Yin et al.'s study supports this, noting higher CHD mortality and risk in males despite a higher baseline proportion of females with excessive sleep (80).

Excessive sleep duration among Asian females associates with CHD risk, potentially exacerbated by psychosocial stressors. Mental health disorders like depression and benzodiazepine use, linked to excessive sleep, are prevalent among Asians who often face long working hours and substantial psychological stress (37, 81–83). Furthermore, the lower rate of psychological treatment in low- and middle-income Asian countries might explain the unique findings in this subgroup (84). This highlights the need for further research to dissect these complex interactions, particularly the non-association of very short sleep durations with CHD risk in North American women, possibly due to limited data inclusion from the region.

Subgroup analysis based on BMI reveals that excessive sleep duration does not correlate with CHD risk in individuals with a BMI ≥24, regardless of gender. This observation aligns with a previous meta-analysis of 40 studies, which noted that while underweight individuals (BMI <20 kg/m^2^) show increased overall and CVD mortality risks, those slightly overweight (BMI: 24–27.9) exhibit the lowest mortality risks. In contrast, severe obesity (BMI ≥35 kg/m^2^) is linked to the highest CVD mortality (85). This suggests that factors other than BMI may influence CHD risk in individuals with a BMI between 24 and 30. Given that only one included study had an average BMI ≥28, further investigation into this category is warranted.

Our findings align with those of Shankar and Sabanayagam (18, 19) but differ from the results by Meisinger, Ikehara, Lao, Yazdanpanah, Zhang, Amagai, Chandola, Svensson, Kronholm, and Strand (21–30). A detailed comparison of adjustment factors in these studies reveals that most studies showing discrepancies did not consider race and working hours as risk factors. Further research is necessary to understand how race and working hours might differently affect genders in terms of CHD risk. Nonetheless, our meta-analysis indicates that both genders face increased CHD risk with abnormal sleep durations, with the risk being particularly pronounced for short sleep durations in women and long durations in men.

Gender-specific sleep interventions are essential based on these differences. For women, maintaining 7.5–8.5 h of daily sleep is recommended, along with stress reduction through yoga and meditation to minimize stress hormone effects, and regular monitoring of blood glucose for early risk detection. For men, optimal sleep duration should be 7.0–8.0 h nightly, complemented by smoking cessation, regular exercise, and sufficient physical activity to reduce weight, improve sleep quality, and prevent obesity and metabolic risks associated with prolonged sleep.

There are three primary limitations to our paper. First, the reliance on self-reported sleep data may introduce bias compared to polysomnography, the gold standard for measuring sleep duration. Second, the number of studies within specific subgroups—such as cross-sectional, case-control, and particularly the North American subgroup—is limited, which may influence the robustness of our conclusions. Third, while our study focuses on sleep duration's correlation with CHD in men and women, some articles did not distinguish CHD from cardiovascular diseases, therefore, a few articles also included stroke patients (19, 26, 29, 34). Nonetheless, these studies passed our sensitivity analysis without issue.

In conclusion, both short and long sleep durations significantly correlate with CHD risk in both genders. Our analysis confirms a significant non-linear U-shaped relationship between sleep duration and CHD risk for both men and women (both *P* < 0.01). Notably, the impact of short sleep duration on CHD risk is more pronounced in women, while long sleep duration more significantly affects men. The optimal sleep duration is identified as 7.0–8.0 h daily for men and 7.5–8.5 h for women. Future longitudinal cohort studies or randomized controlled trials should evaluate the associations between sleep duration and CHD across different regions, age groups, and BMI.

## Data Availability

The original contributions presented in the study are included in the article/Supplementary Material, further inquiries can be directed to the corresponding author.
