# Supplementary material for: Gender correlation between sleep duration and risk of coronary heart disease: a systematic review and meta-analysis
Source: Front Cardiovasc Med. 2025 Mar 25;12:1452006. doi: 10.3389/fcvm.2025.1452006 (PMC11975931; doi:10.3389/fcvm.2025.1452006)
Supplement: Supplementary file 1 [file Datasheet1.docx]

**
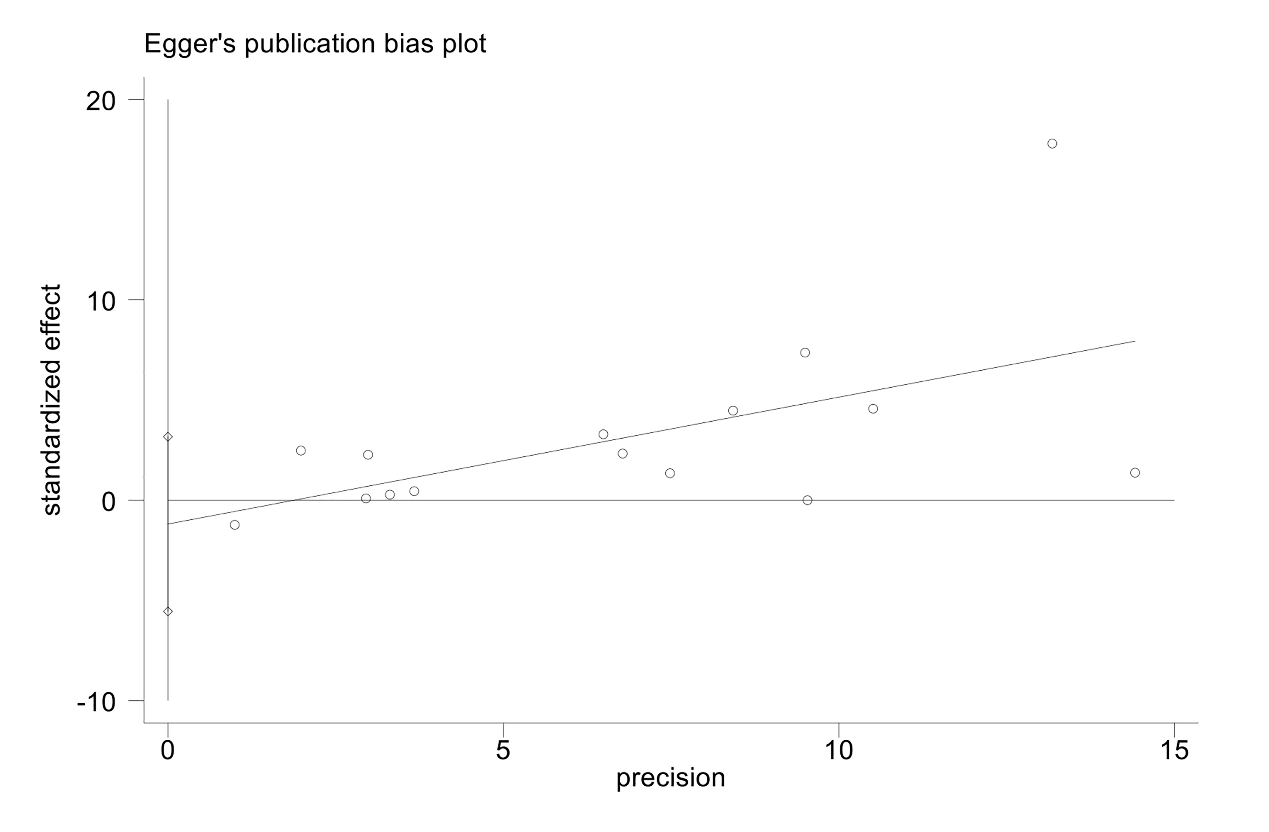
**

**Figure S1:** Publication biased results of short sleep duration and risk of coronary heart disease in men.


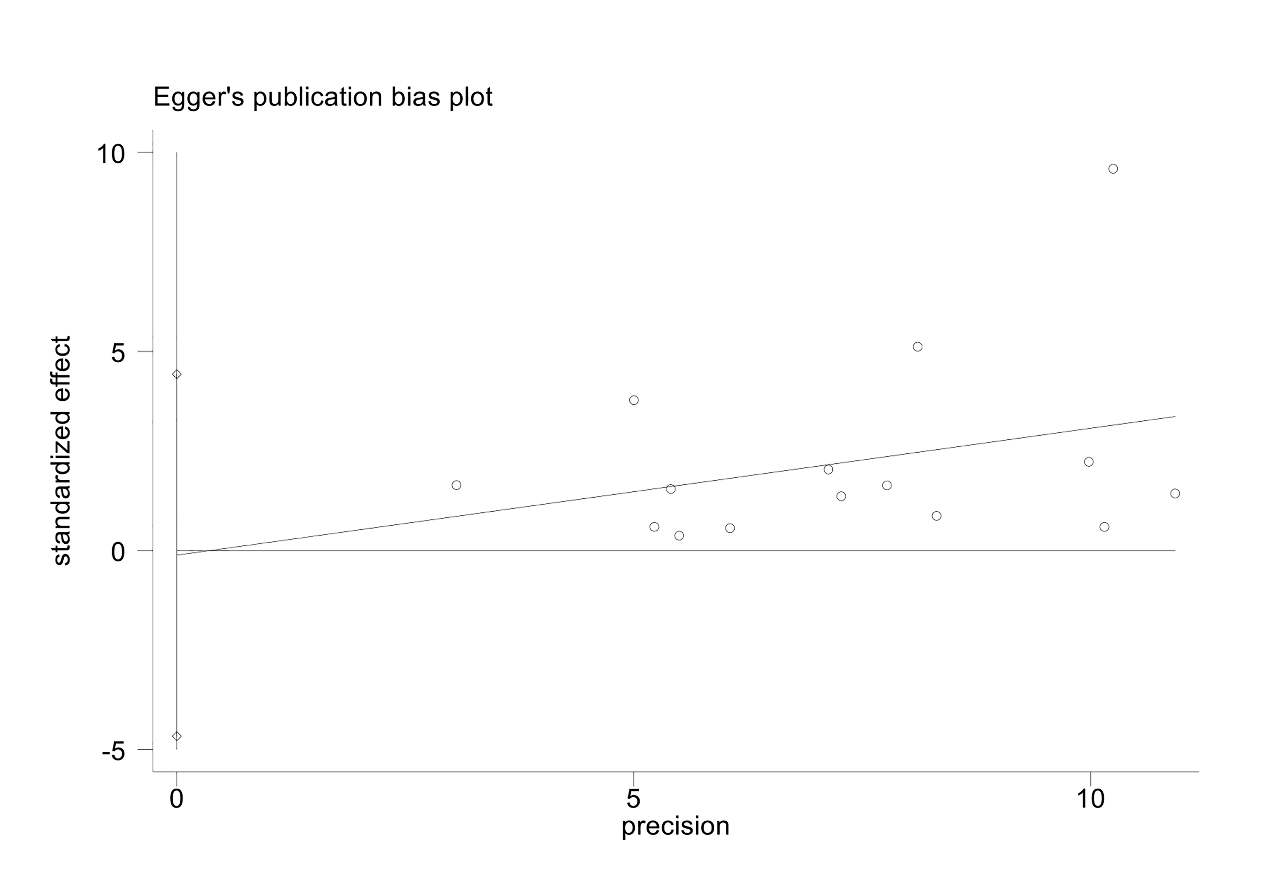


**Figure S2:** Publication biased results of long sleep duration and risk of coronary heart disease in men.


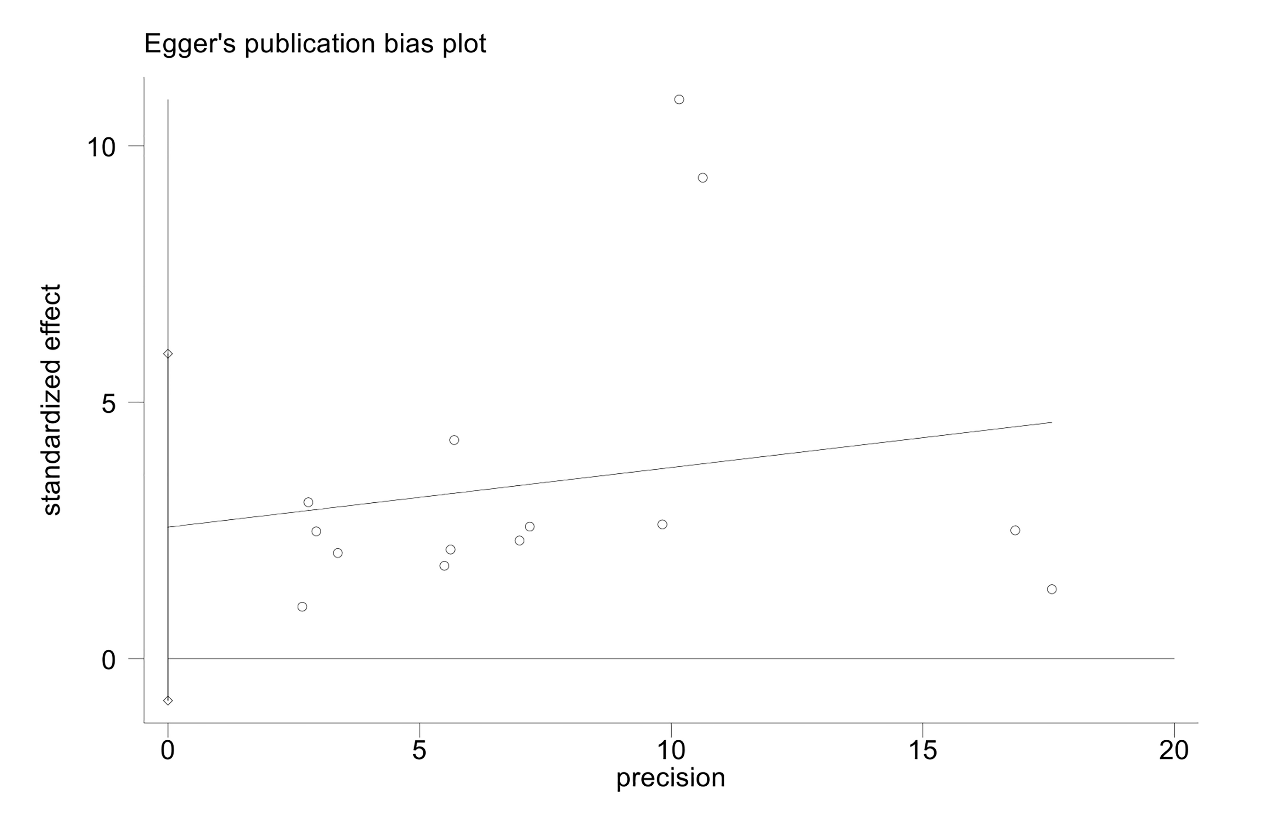


**Figure S3:** Publication biased results of short sleep duration and risk of coronary heart disease in women.


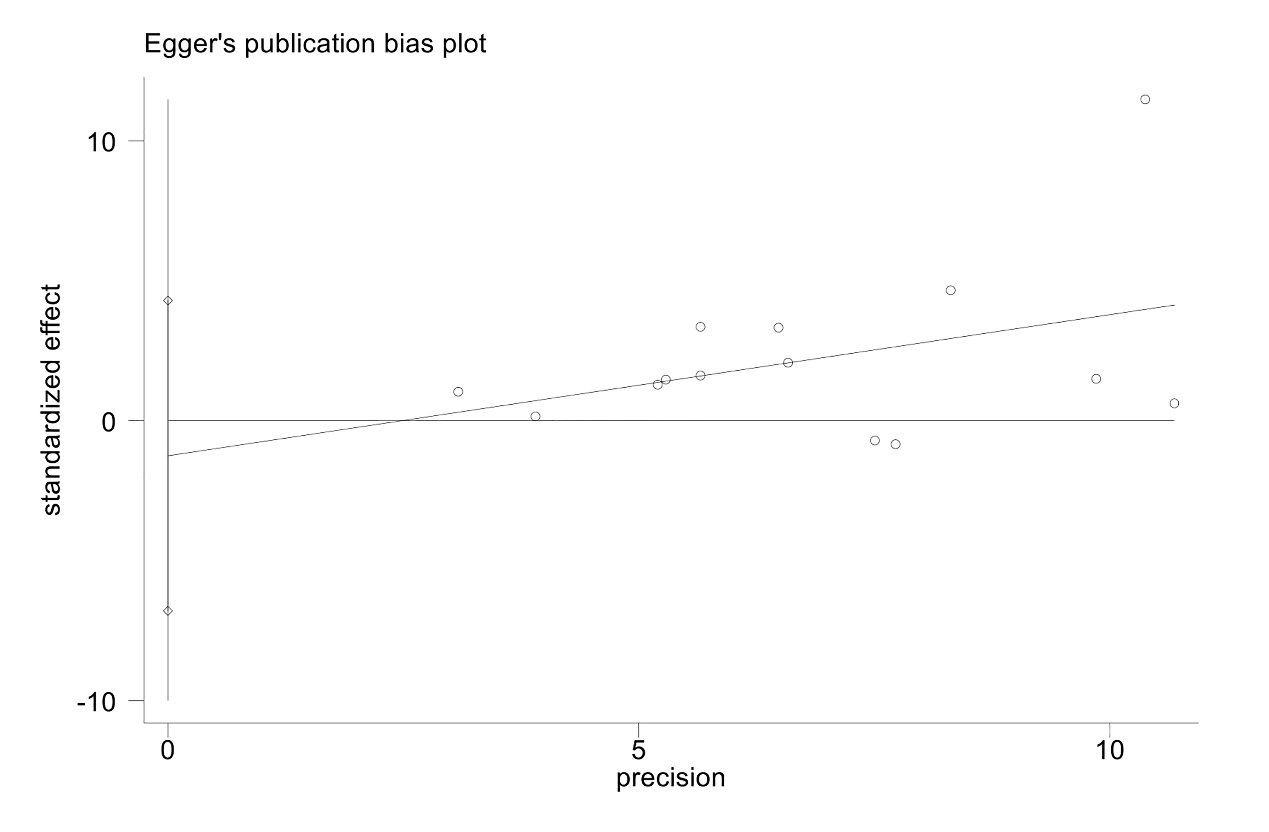


**Figure S4:** Publication biased results of long sleep duration and risk of coronary heart disease in women.


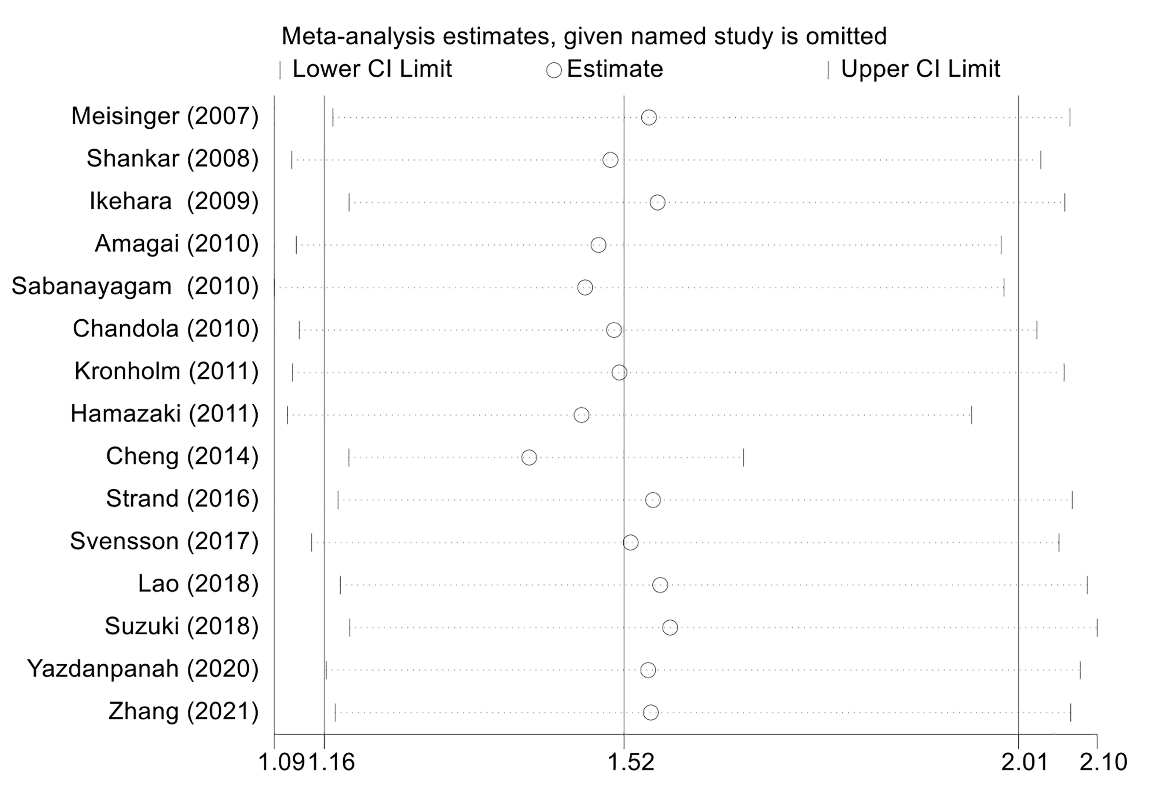


**Figure S5:** Sensitivity analysis of short sleep duration and risk of coronary heart disease in men.


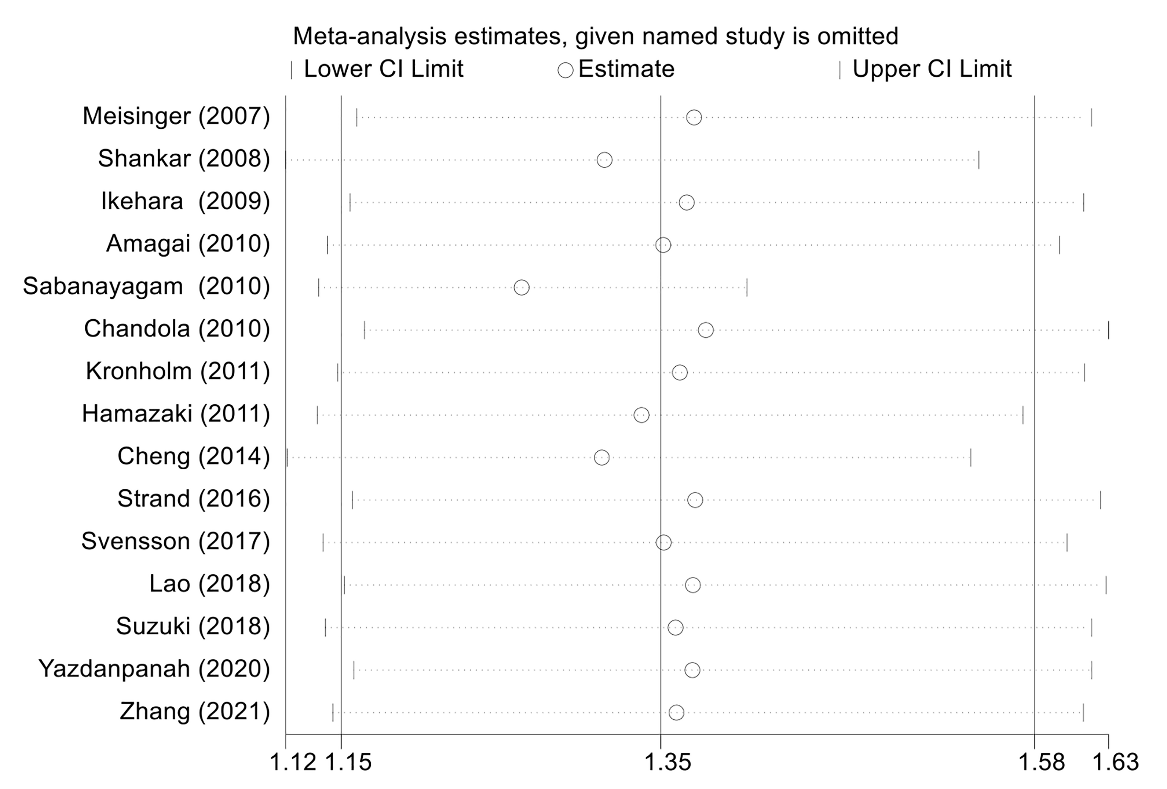


**Figure S6:** Sensitivity analysis of long sleep duration and risk of coronary heart disease in men.


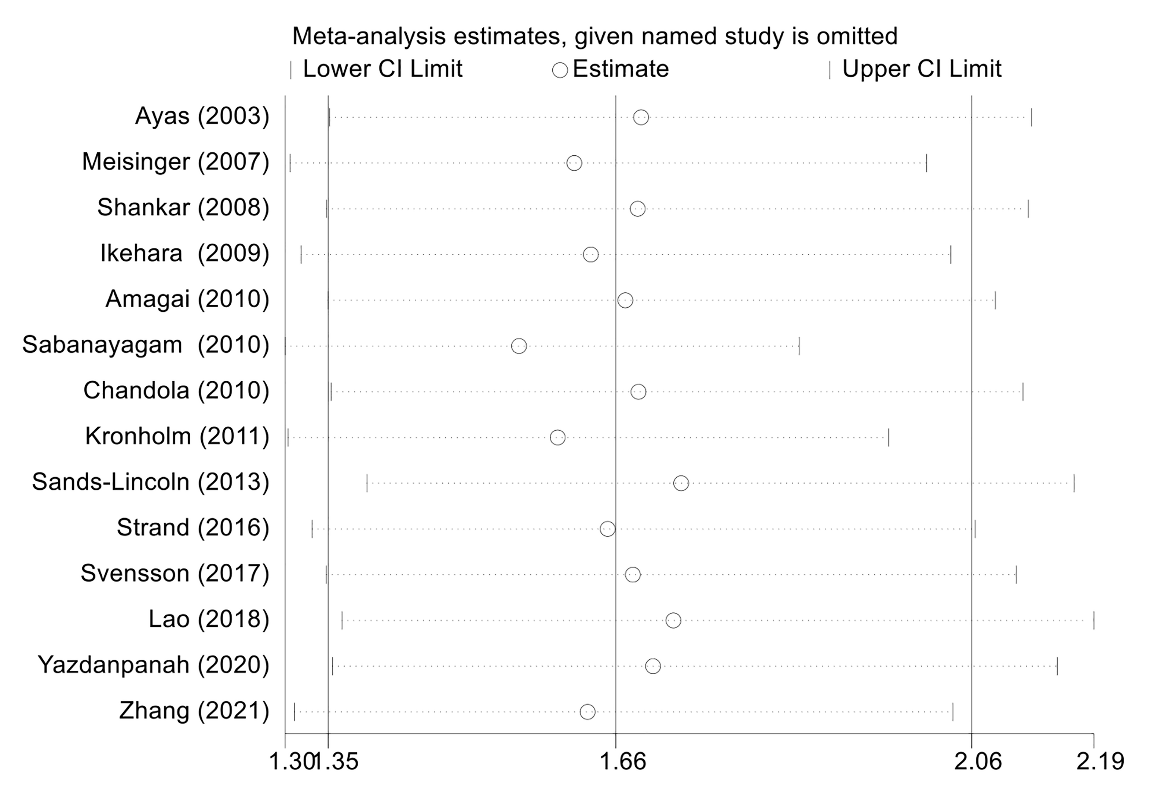


**Figure S7:** Sensitivity analysis of short sleep duration and risk of coronary heart disease in women.


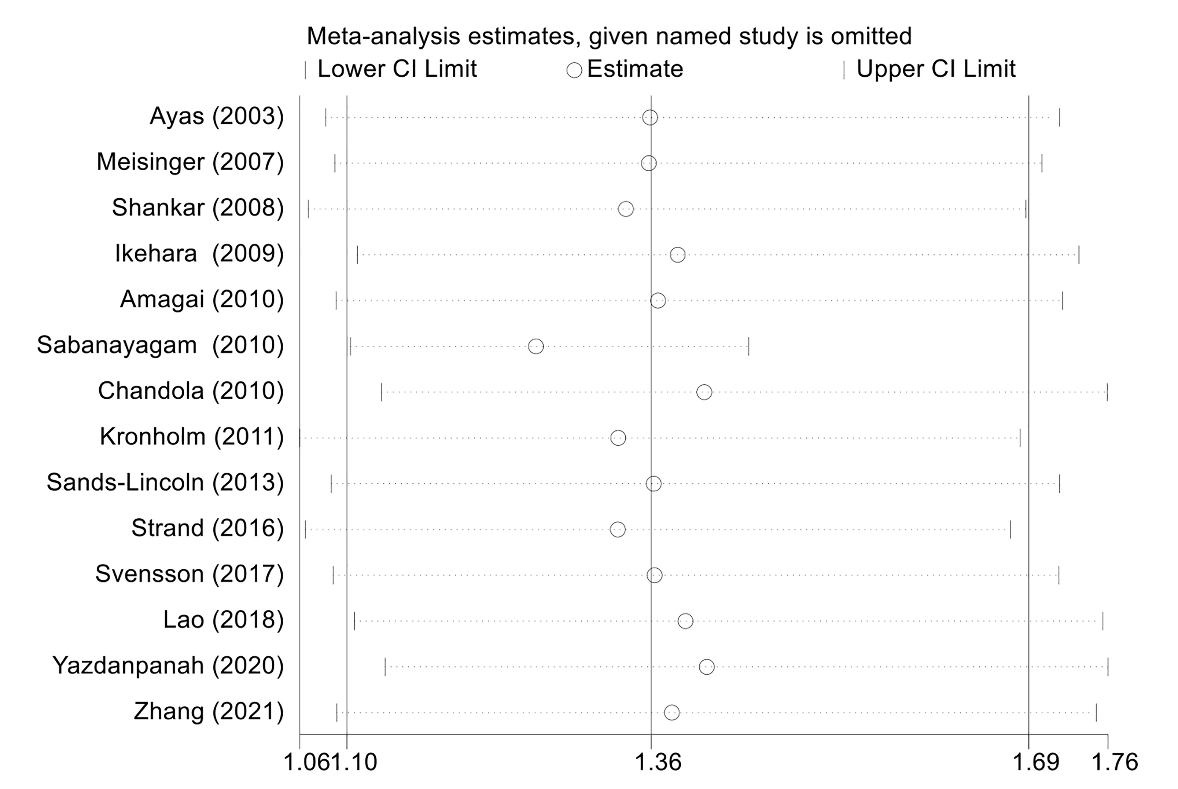


**Figure S8:** Sensitivity analysis of long sleep duration and risk of coronary heart disease in women.


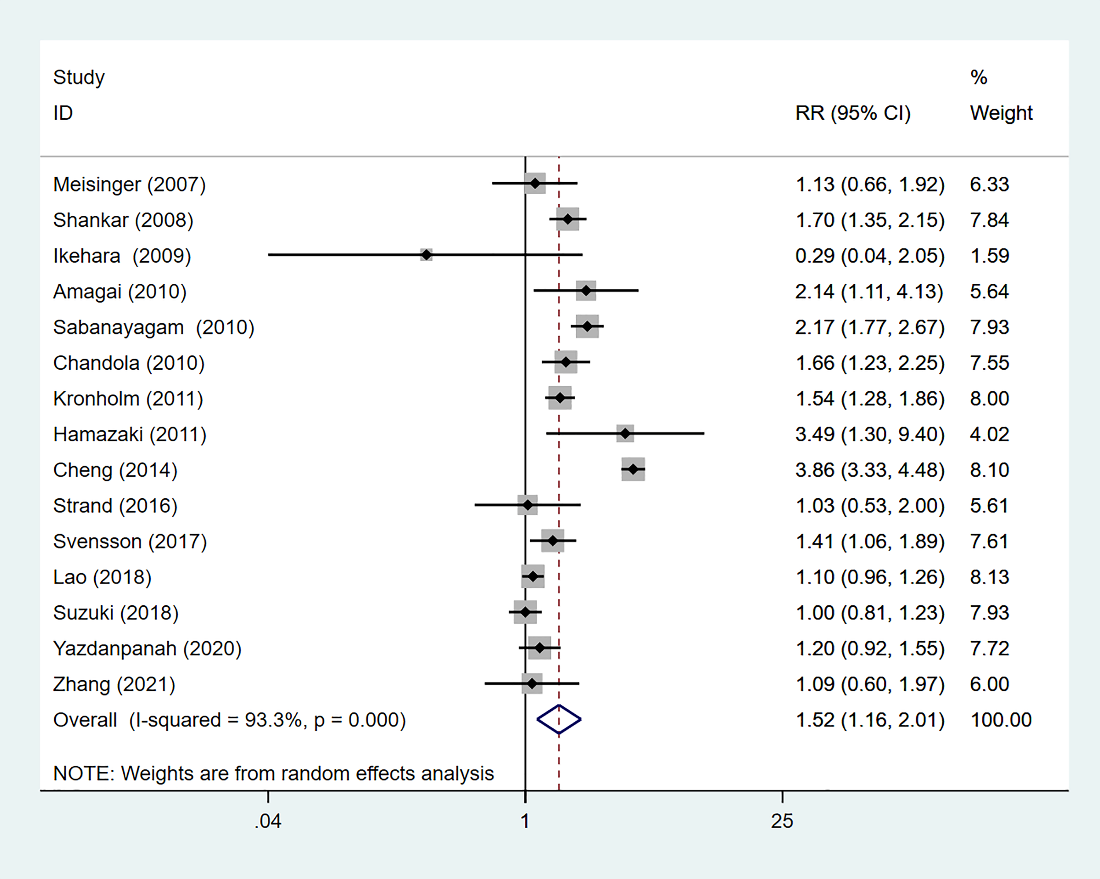


**Figure S9:** Forest plot of RR of the association between short sleep duration and risk of CHD in men.


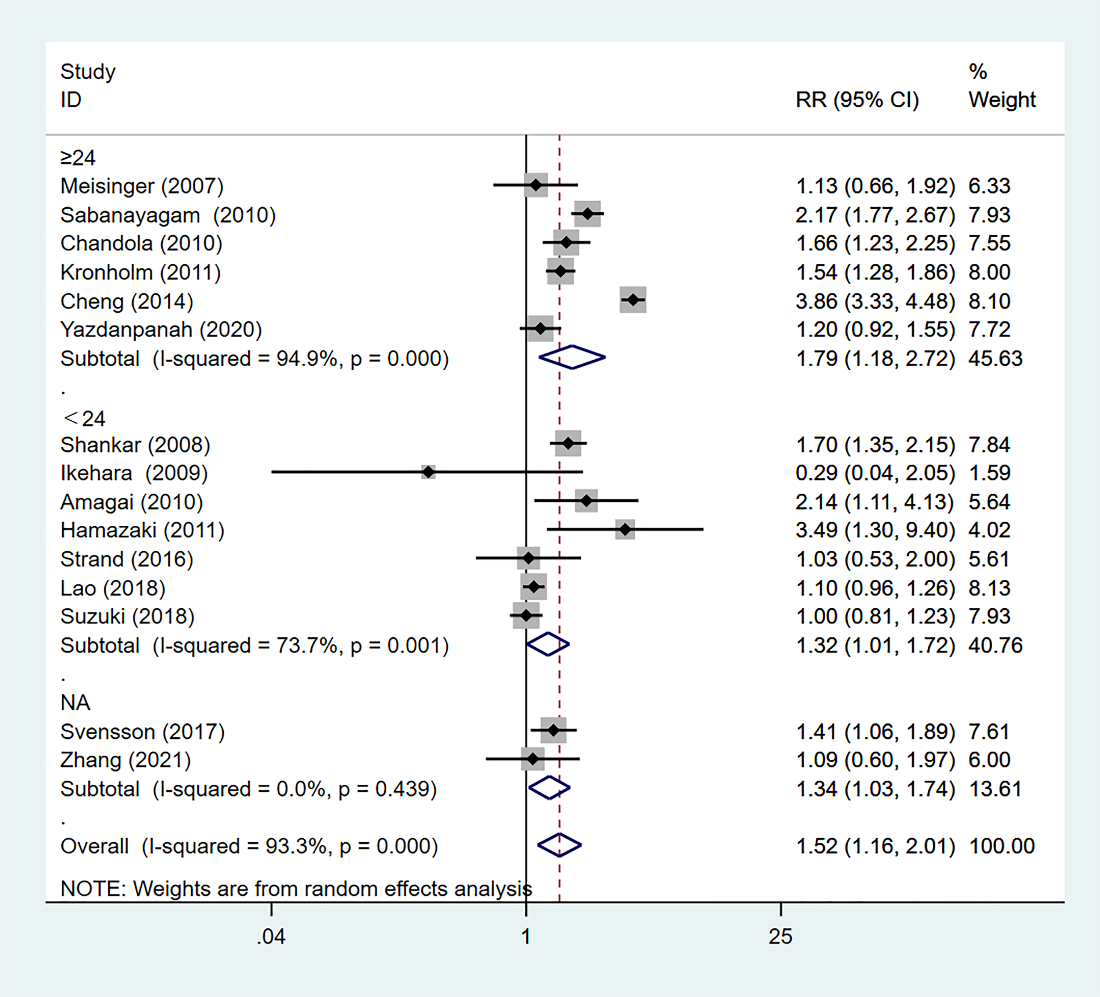


**Figure S10:** Average BMI subgroup analysis of short sleep duration and risk of CHD in men.


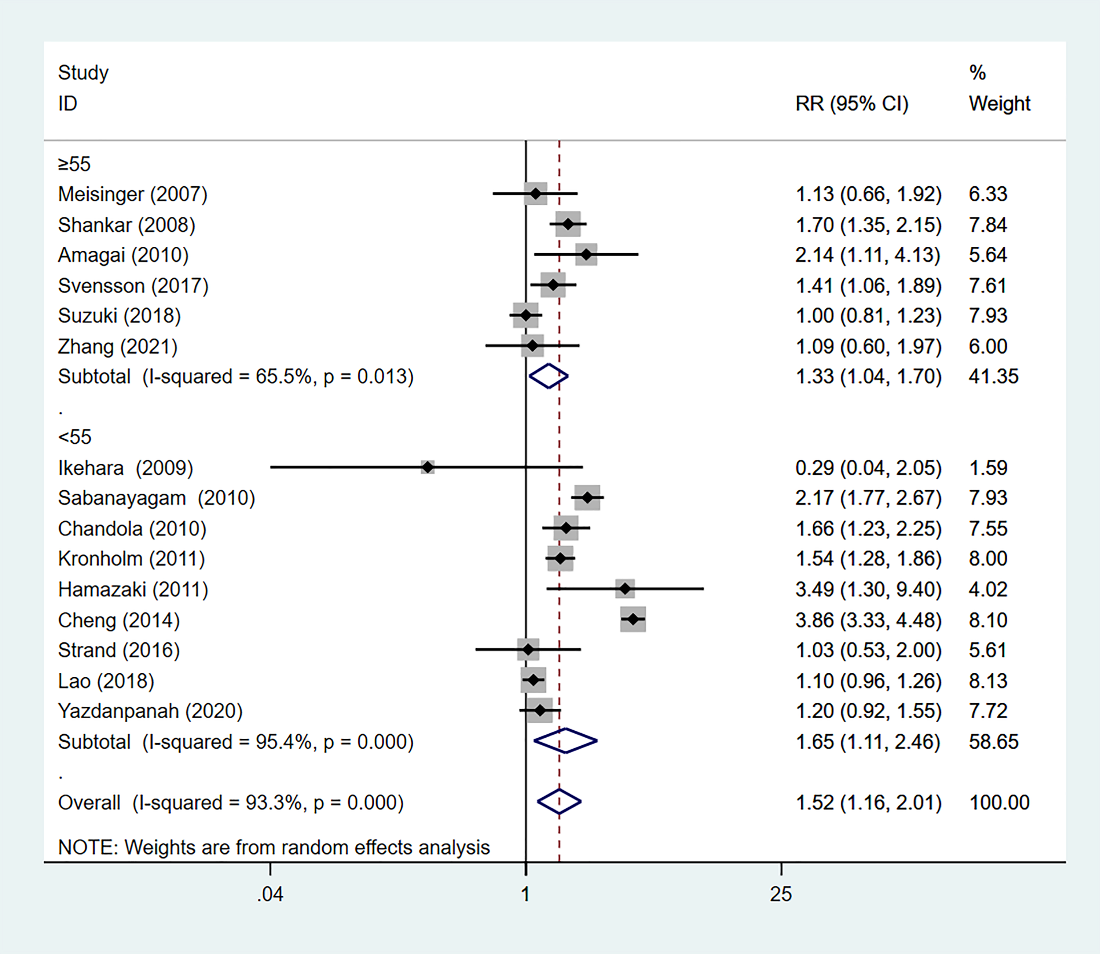


**Figure S11:** Mean age subgroup analysis of short sleep duration and risk of CHD in men.


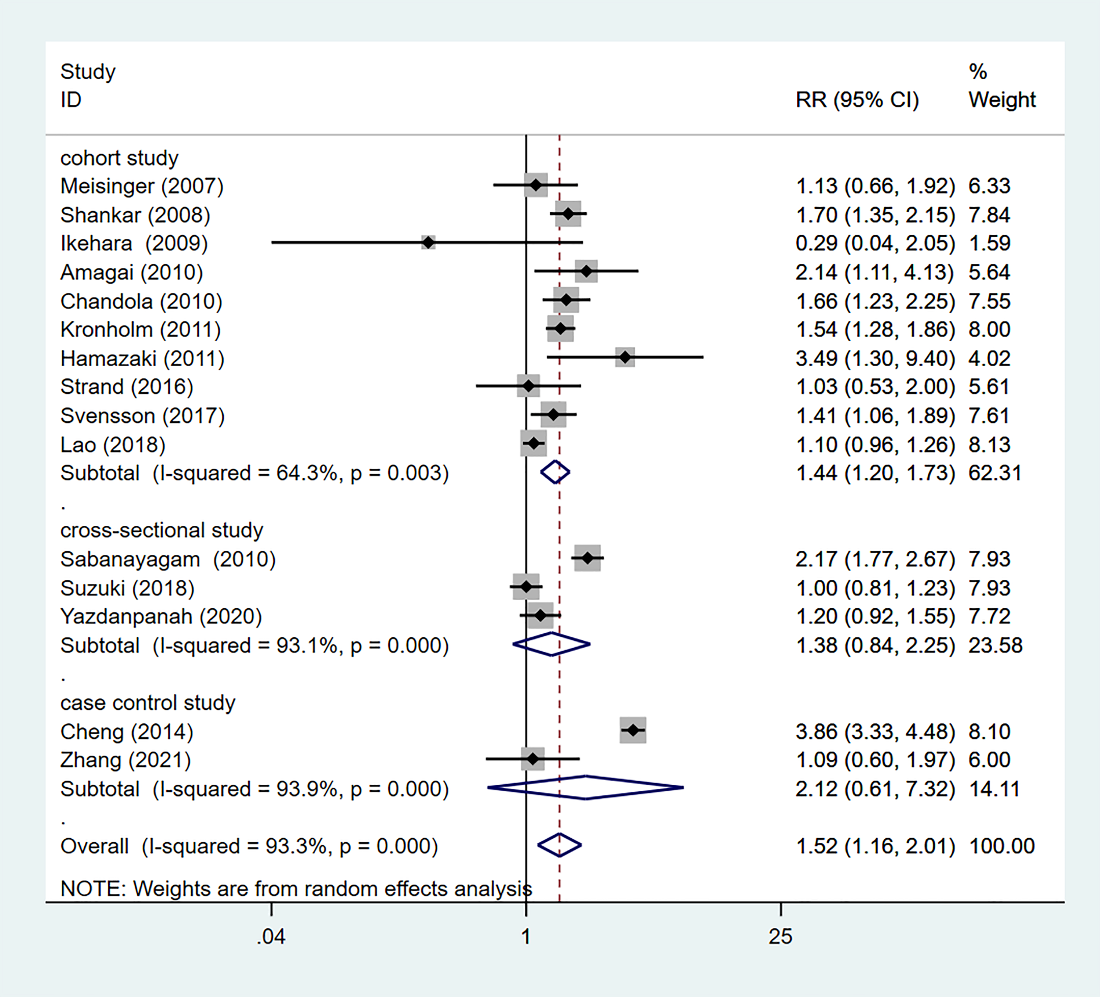


**Figure S12:** Study type subgroup analysis of short sleep duration and risk of CHD in men.


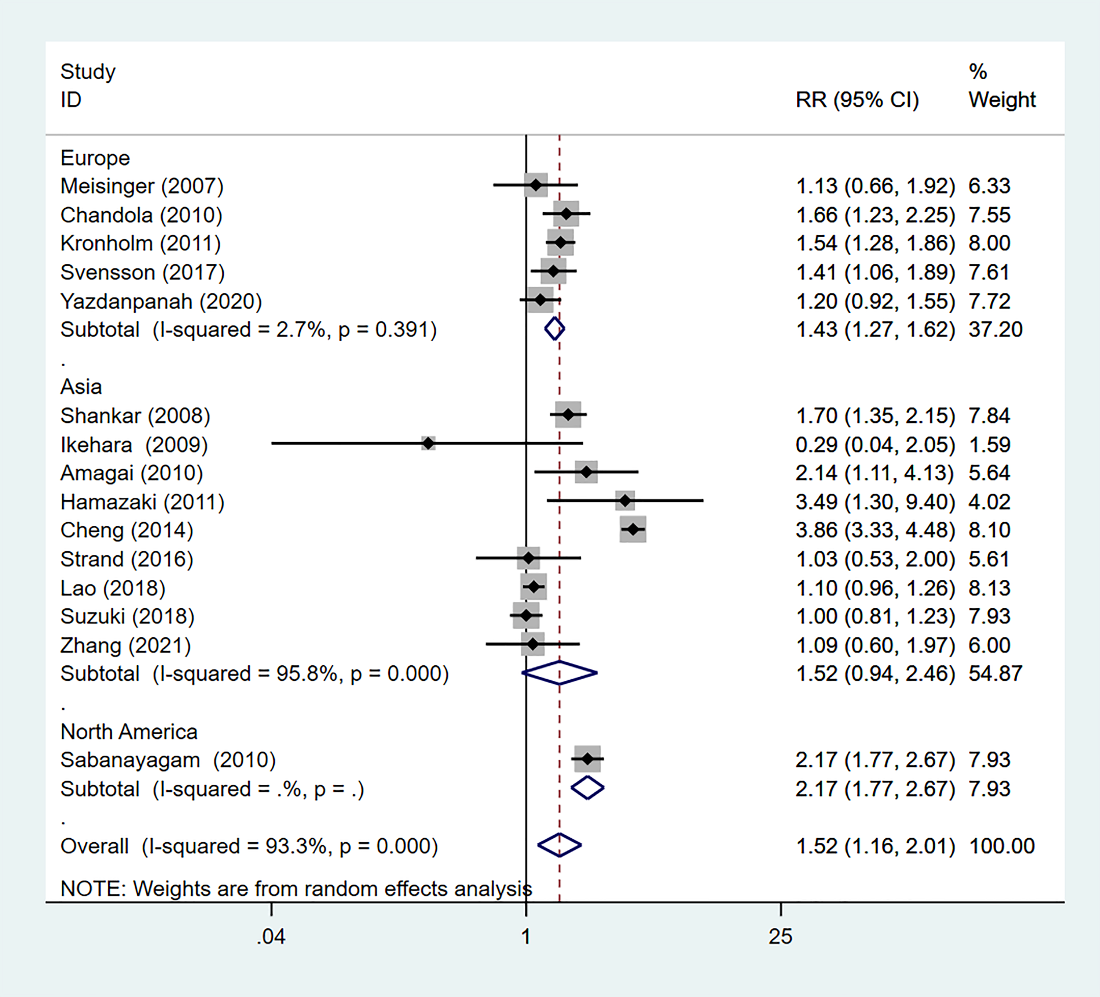


**Figure S13:** Regional subgroup analysis of short sleep duration and risk of CHD in men.


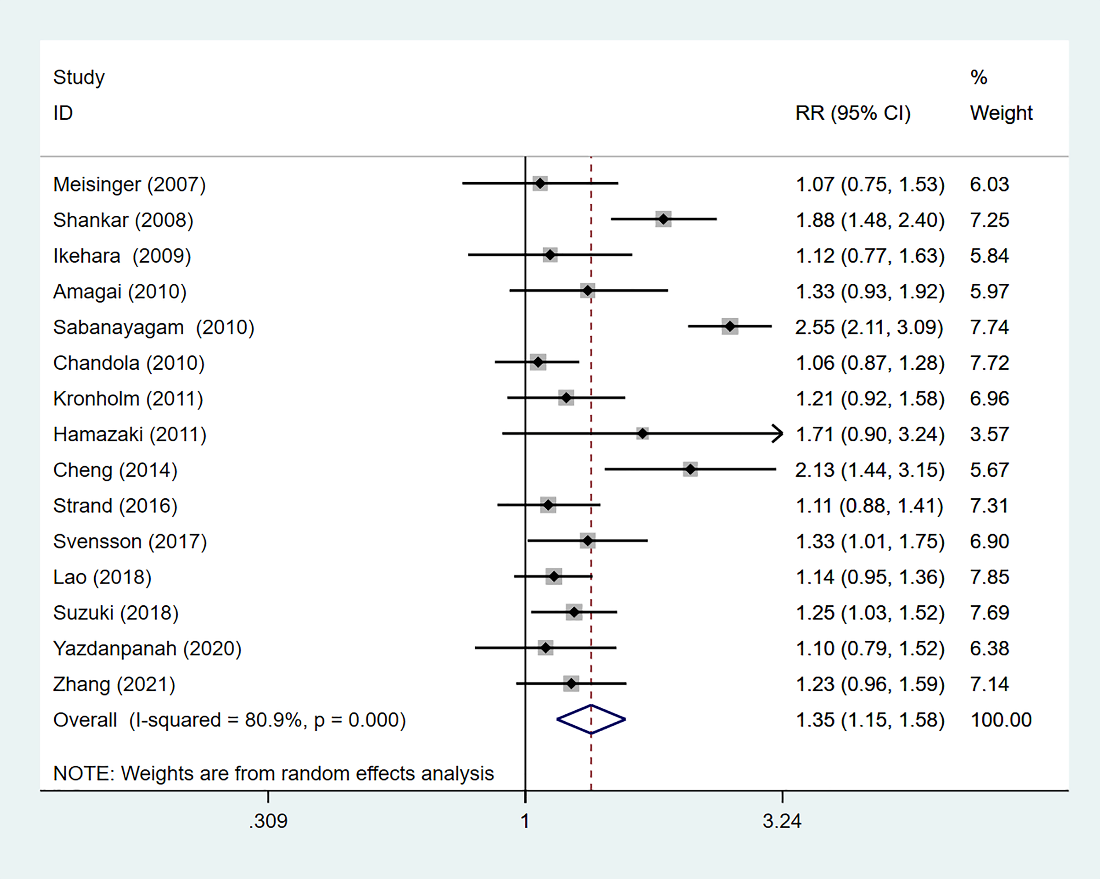


**Figure S14:** Forest plot of RR of the association between long sleep duration and risk of CHD in men.


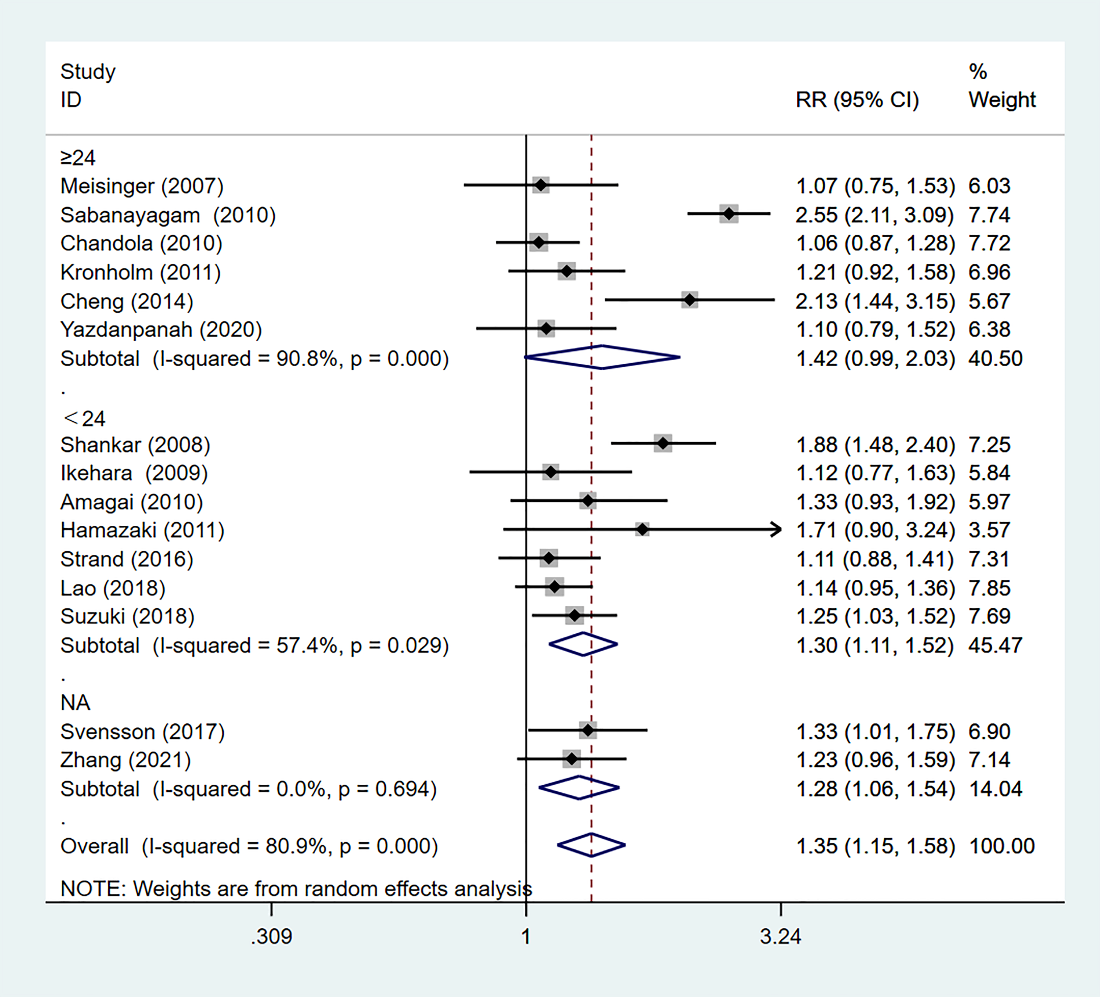


**Figure S15:** Average BMI subgroup analysis of long sleep duration and risk of CHD in men.


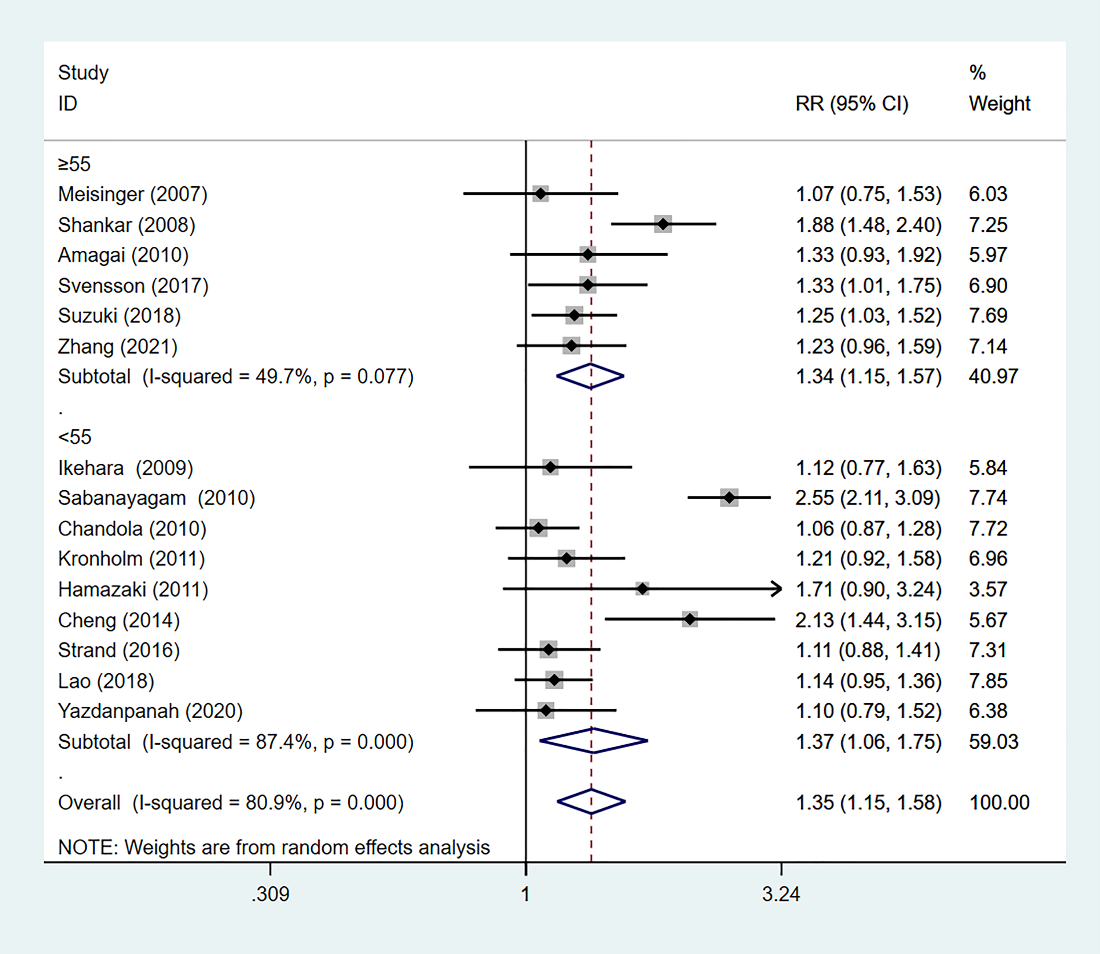


**Figure S16:** Mean age subgroup analysis of long sleep duration and risk of CHD in men.


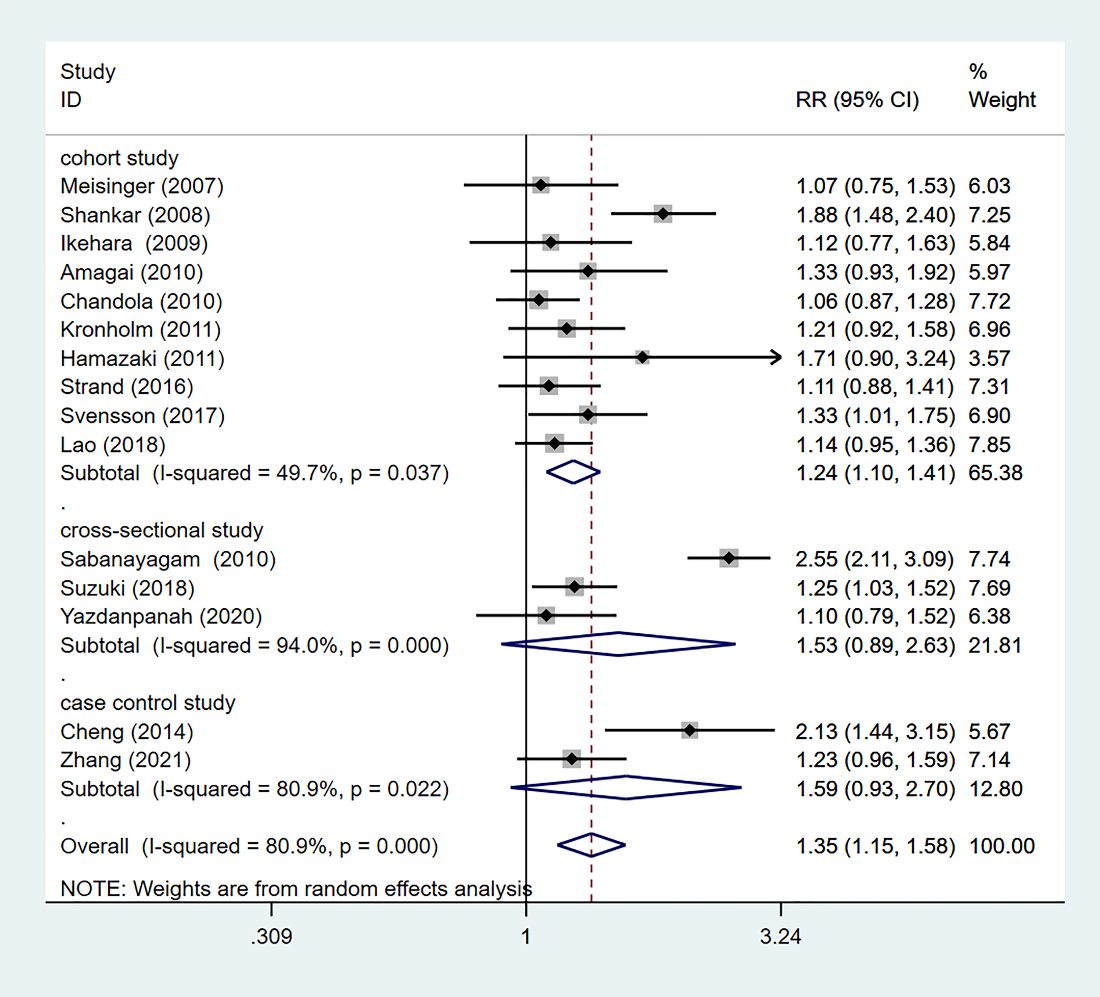


**Figure S17:** Study type subgroup analysis of long sleep duration and risk of CHD in men.


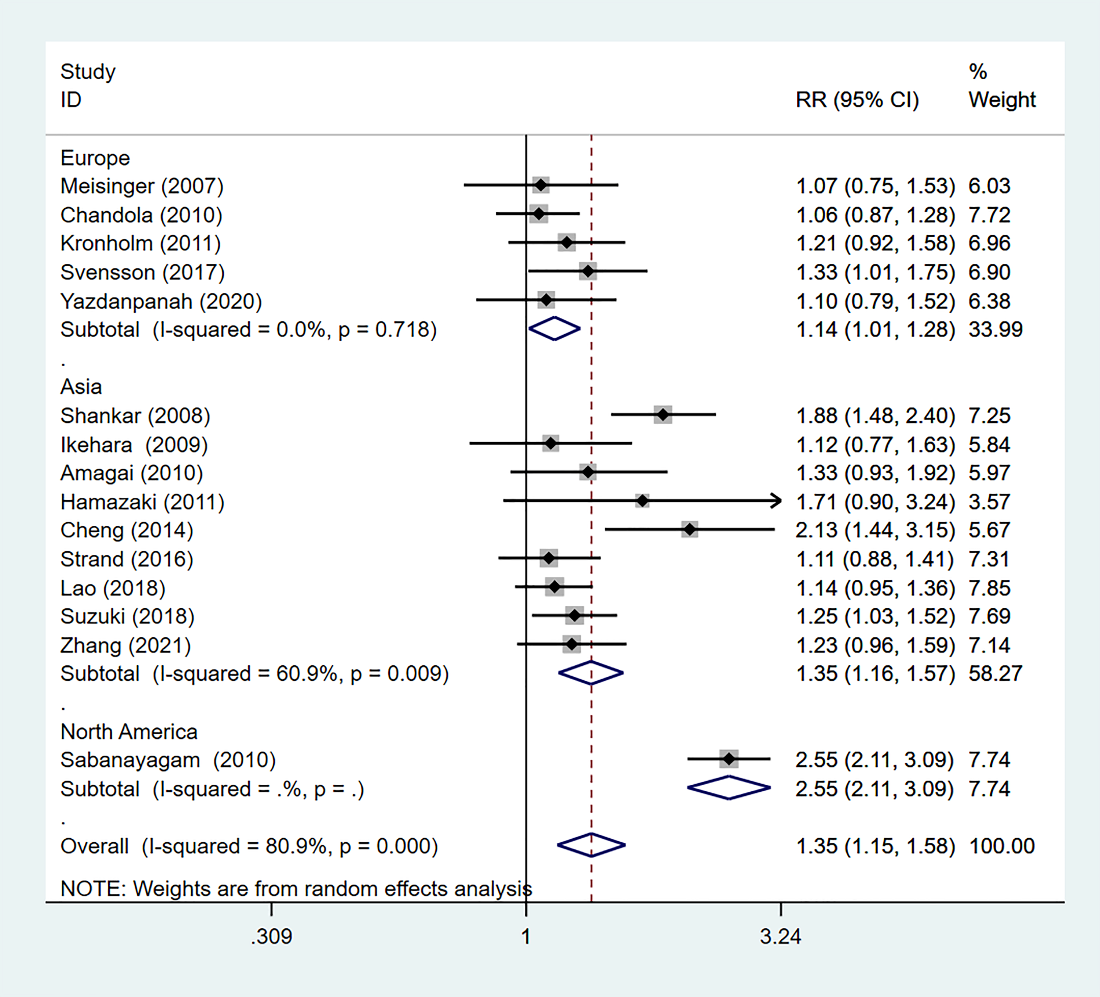


**Figure S18:** Regional subgroup analysis of long sleep duration and risk of CHD in men.


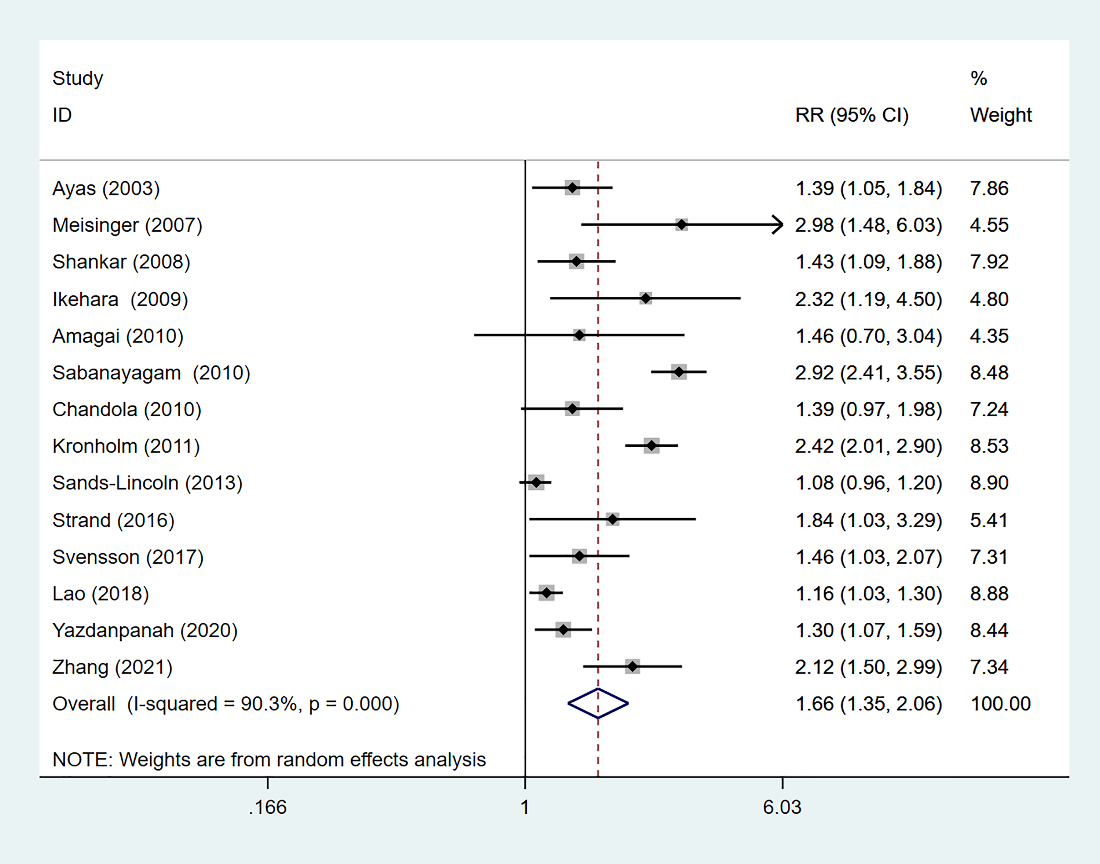


**Figure S19:** Forest plot of RR of the association between short sleep duration and risk of CHD in women.


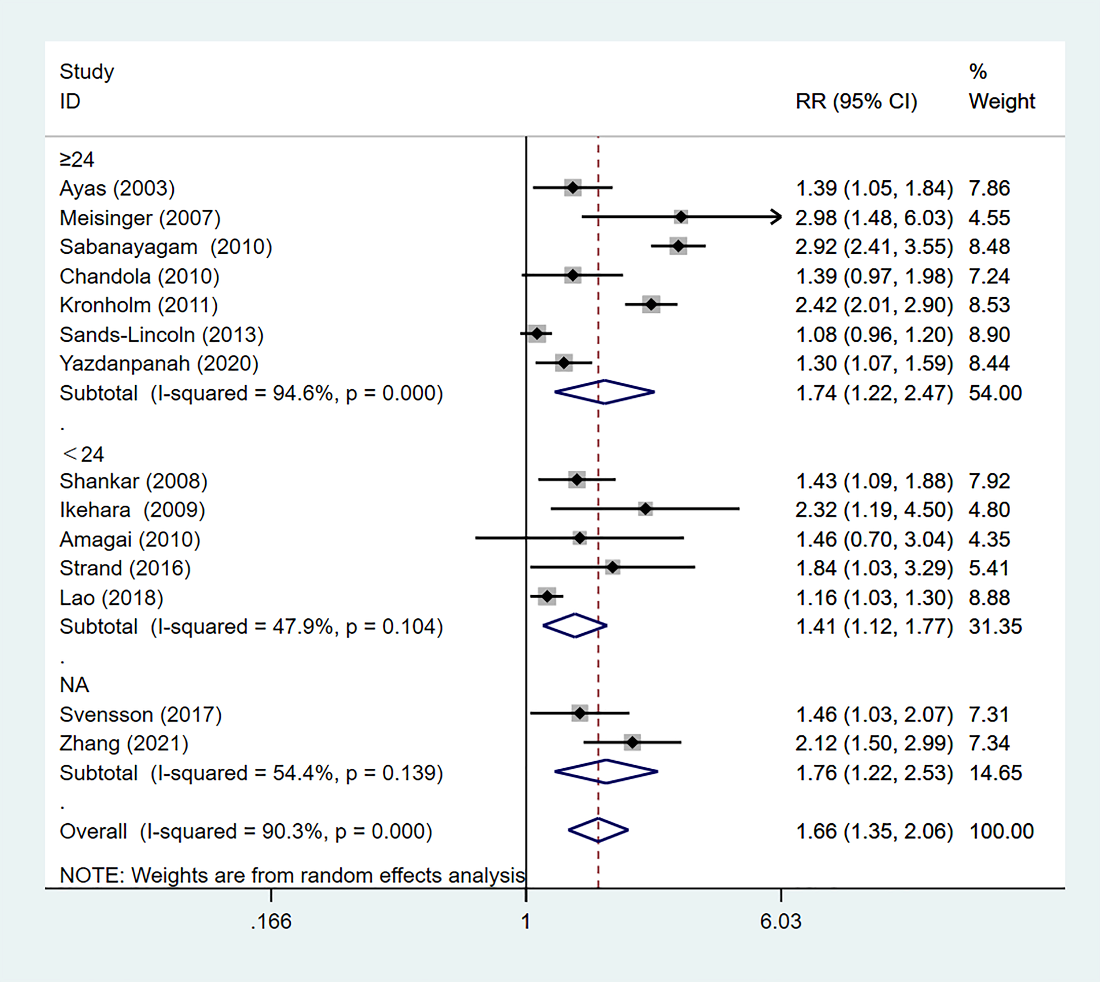


**Figure S20:** Average BMI subgroup analysis of short sleep duration and risk of CHD in women.


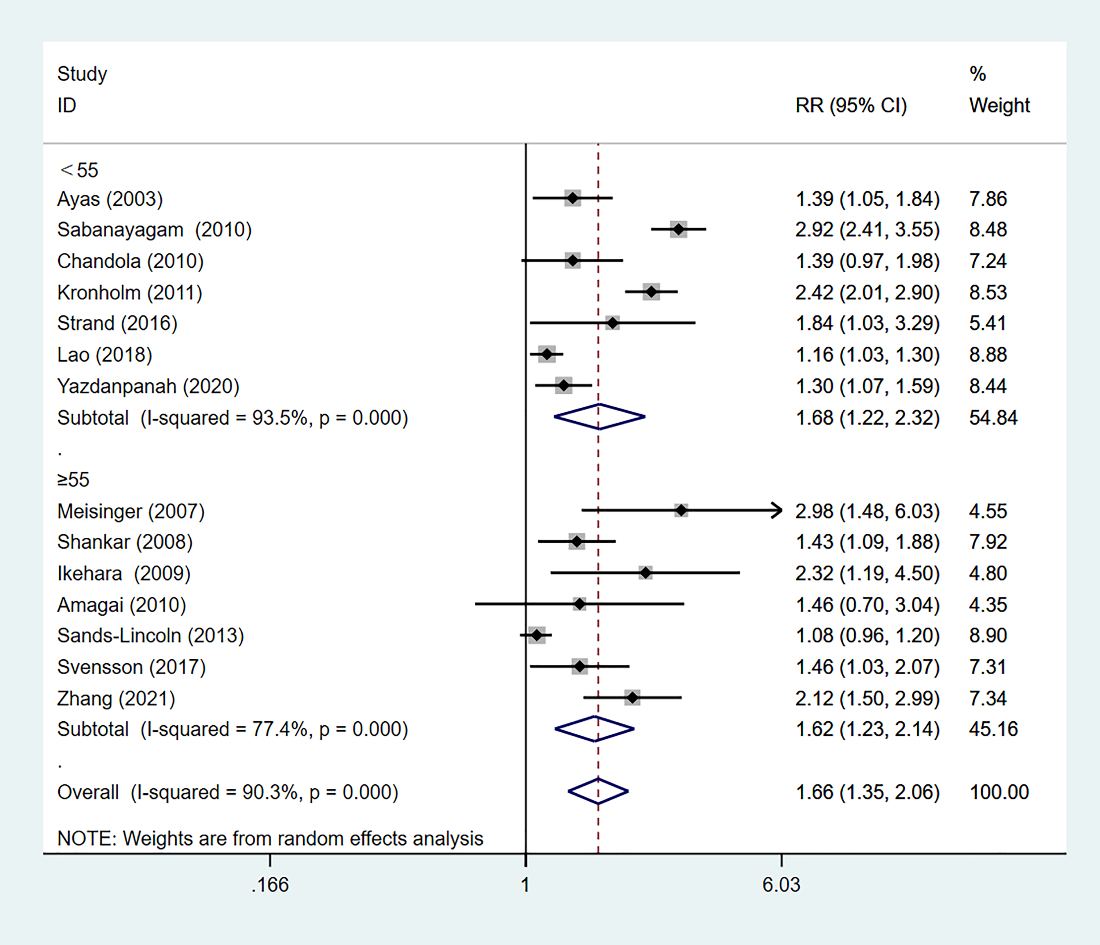


**Figure S21:** Mean age subgroup analysis of short sleep duration and risk of CHD in women.


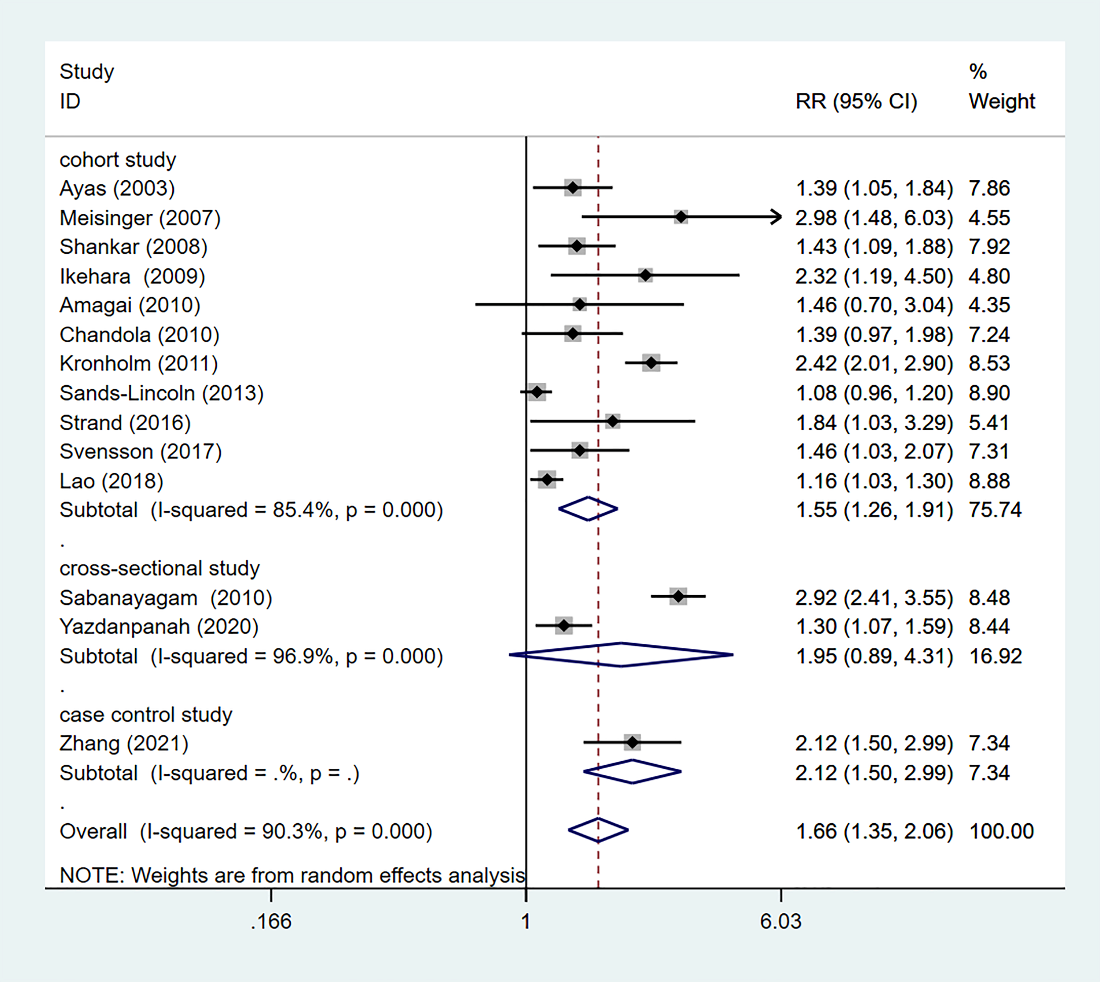


**Figure S22:** Study type subgroup analysis of short sleep duration and risk of CHD in women.


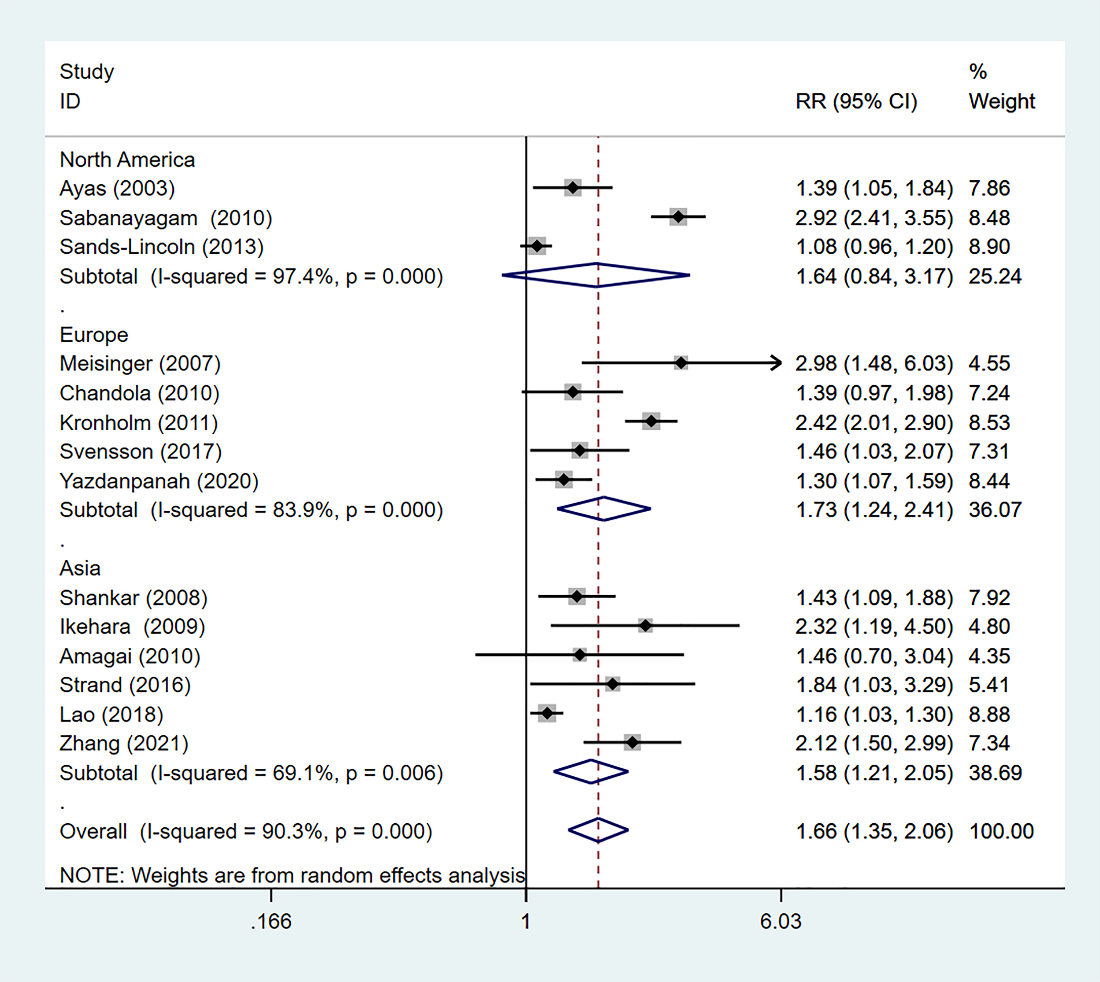


**Figure S23:** Regional subgroup analysis of short sleep duration and risk of CHD in women.


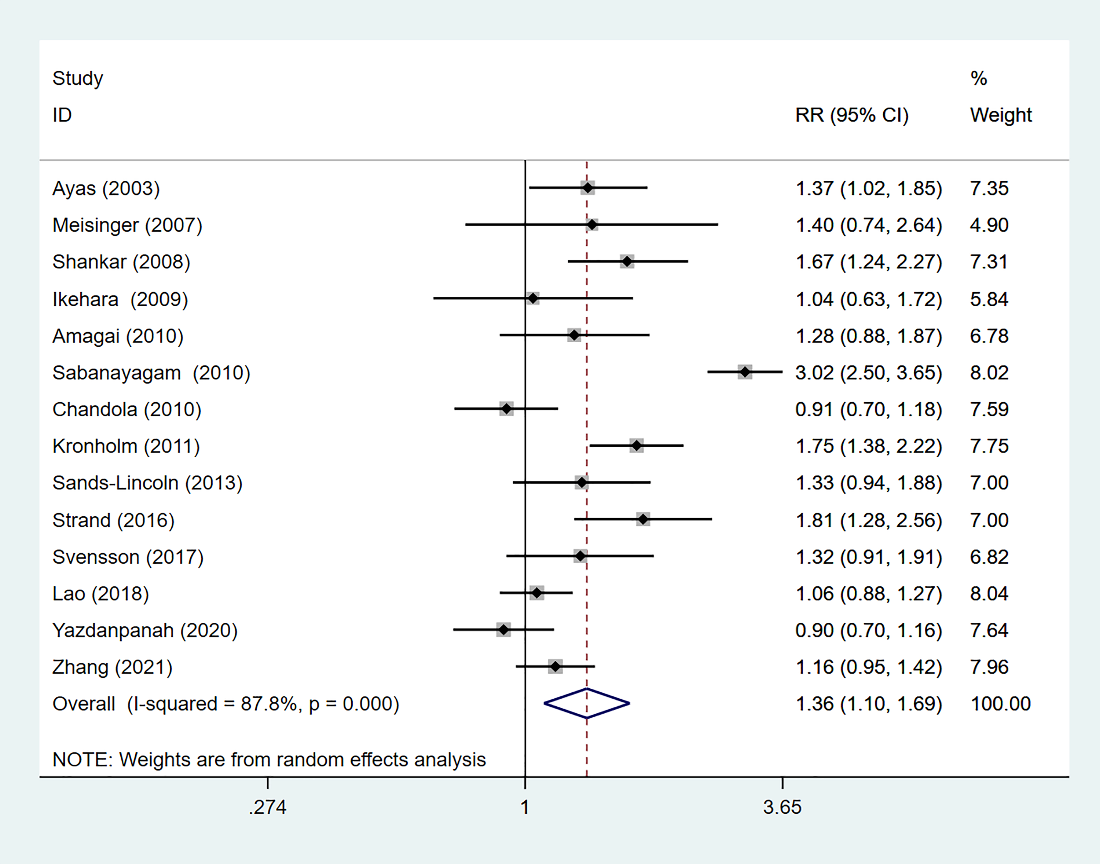


**Figure S24:** Forest plot of RR of the association between long sleep duration and risk of CHD in women.


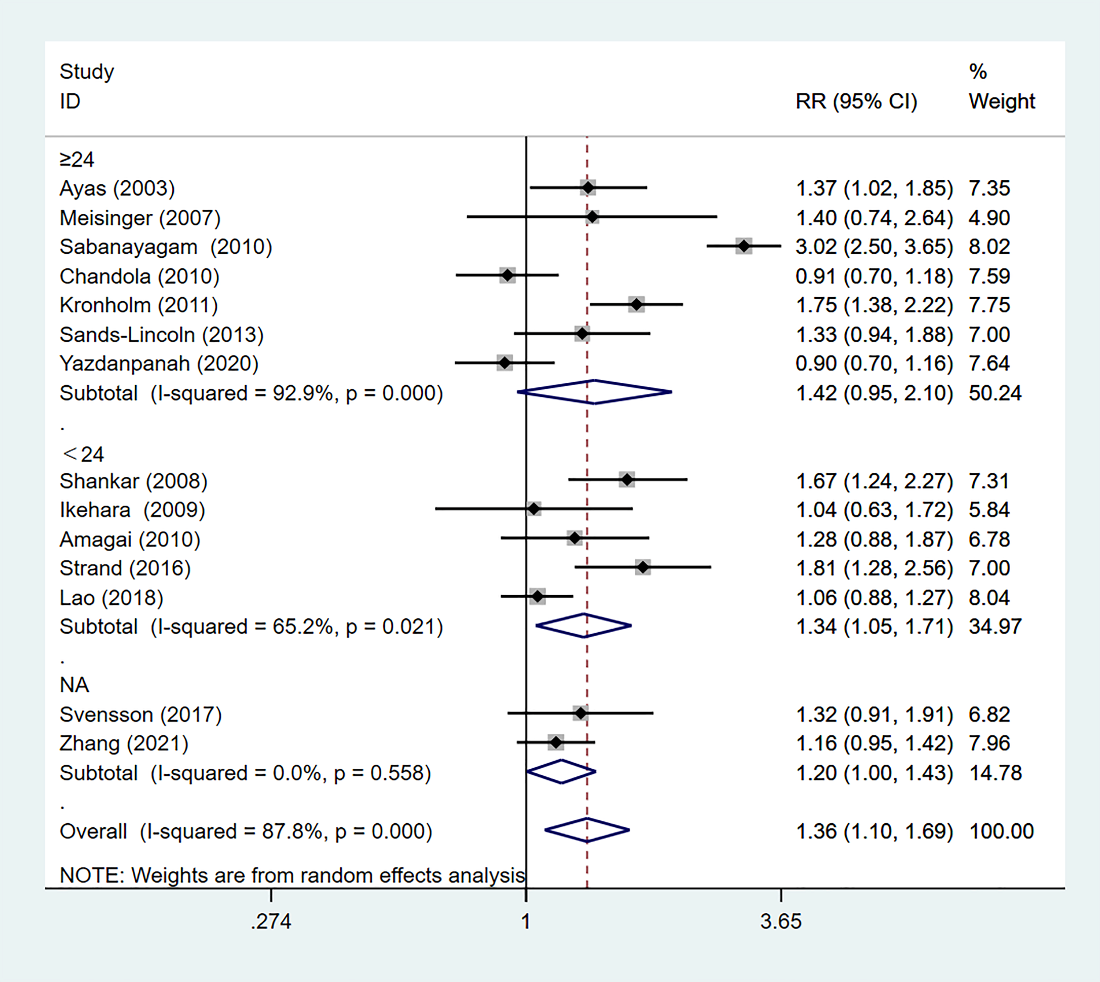


**Figure S25:** Average BMI subgroup analysis of long sleep duration and risk of CHD in women.


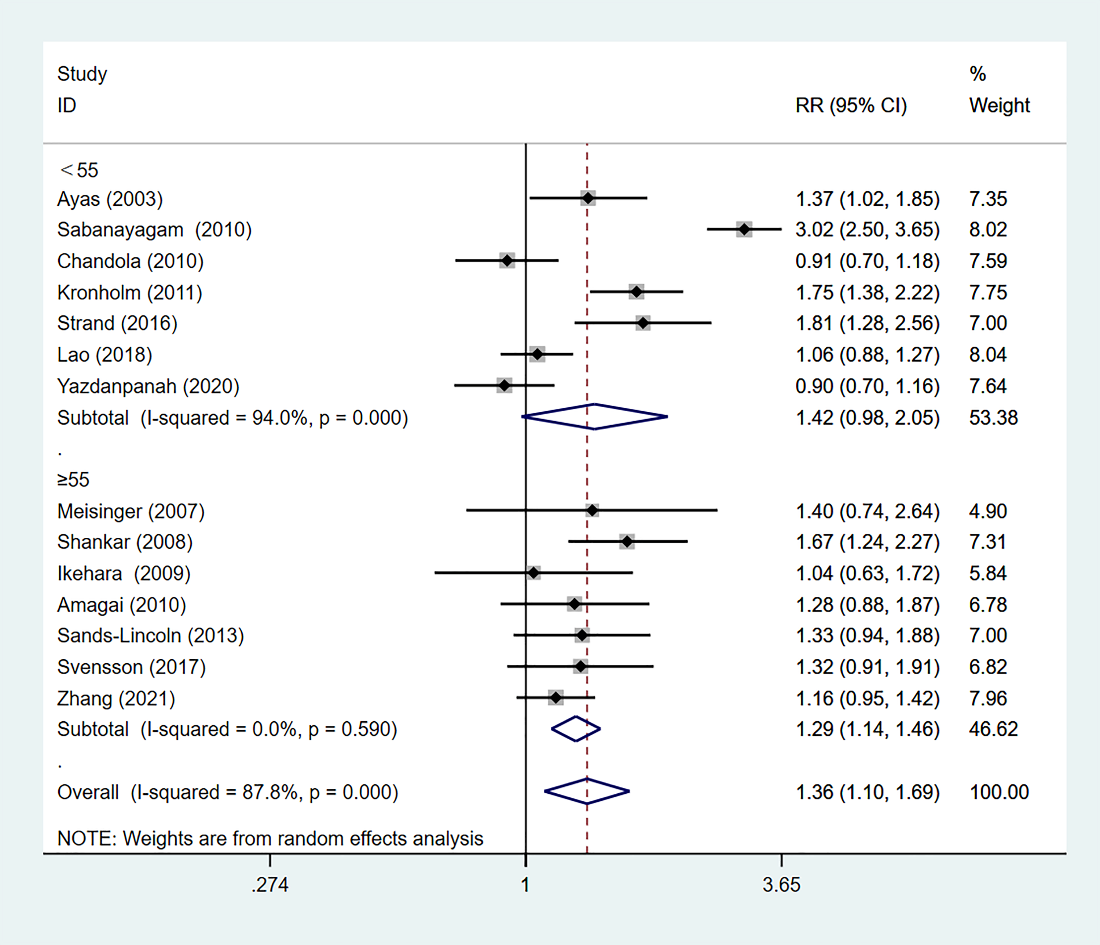


**Figure S26:** Mean age subgroup analysis of long sleep duration and risk of CHD in women.


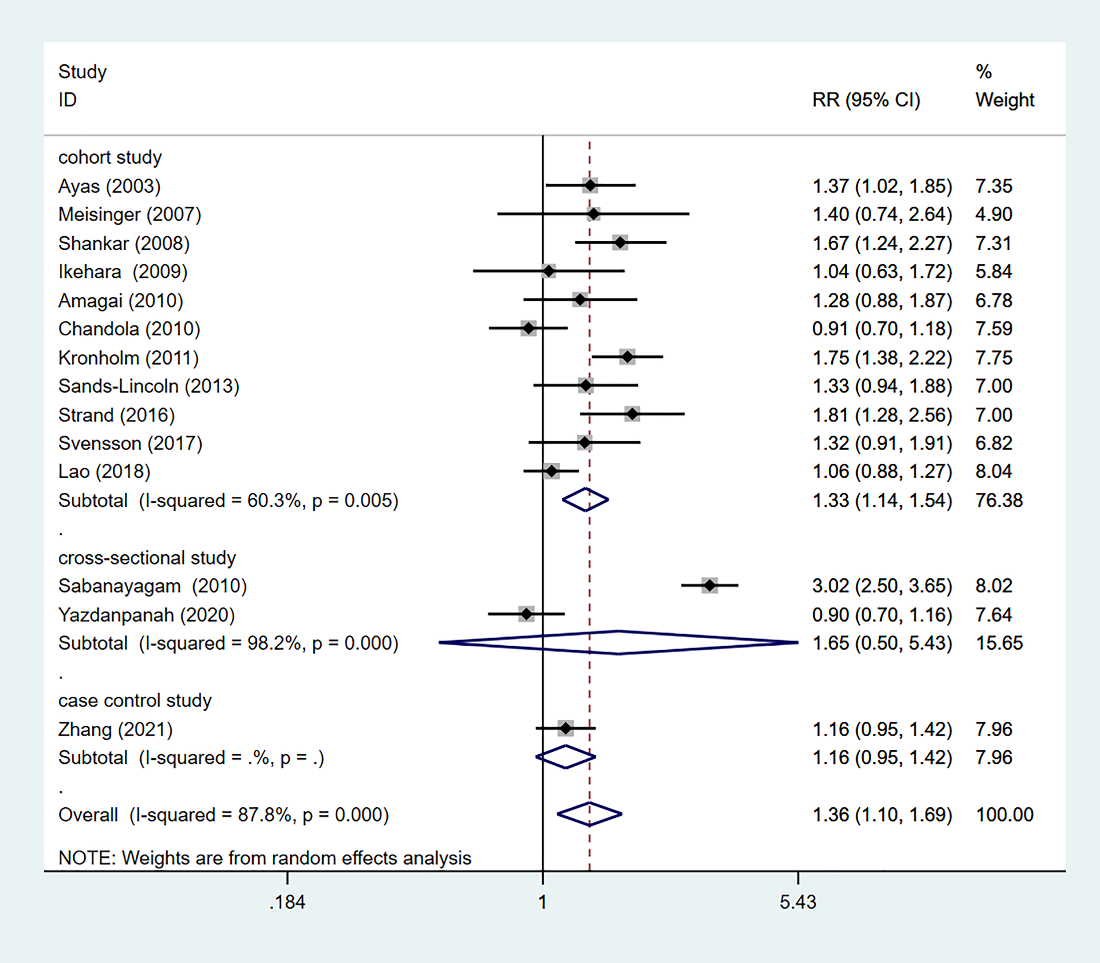


**Figure S27:** Study type subgroup analysis of long sleep duration and risk of CHD in women.


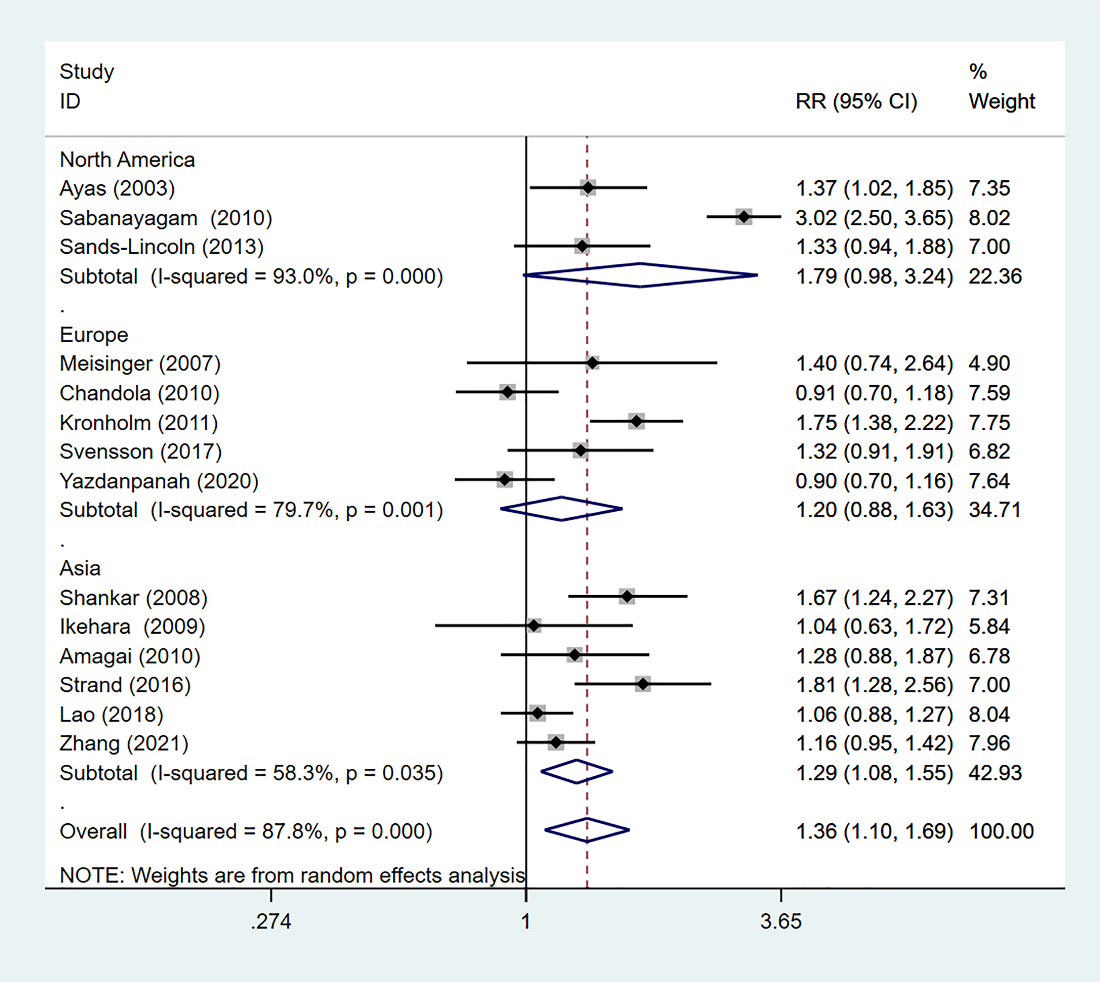


**Figure S28:** Regional subgroup analysis of long sleep duration and risk of CHD in women.
